# Genome-wide association study identifies a missense variant at *APOA5* for coronary artery disease in Multi-Ethnic Cohorts from Southeast Asia

**DOI:** 10.1038/s41598-017-18214-z

**Published:** 2017-12-20

**Authors:** Yi Han, Rajkumar Dorajoo, Xuling Chang, Ling Wang, Chiea-Chuen Khor, Xueling Sim, Ching-Yu Cheng, Yuan Shi, Yih Chung Tham, Wanting Zhao, Miao Ling Chee, Charumathi Sabanayagam, Miao Li Chee, Nicholas Tan, Tien Yin Wong, E-Shyong Tai, Jianjun Liu, Daniel Y. T. Goh, Jian-Min Yuan, Woon-Puay Koh, Rob M. van Dam, Adrian F. Low, Mark Yan-Yee Chan, Yechiel Friedlander, Chew-Kiat Heng

**Affiliations:** 1 Department of Paediatrics, Yong Loo Lin School of Medicine, National University of Singapore; and Khoo Teck Puat - National University Children’s Medical Institute, National University Health System, Singapore, Singapore; 2 Genome Institute of Singapore, Agency for Science, Technology and Research, Singapore, Singapore; 3 Saw Swee Hock School of Public Health, National University of Singapore and National University Health System, Singapore, Singapore; 4 Singapore Eye Research Institute, Singapore National Eye Centre, Singapore, Singapore; 5 Ophthalmology & Visual Sciences Academic Clinical Program (Eye ACP), Duke-NUS Medical School, Singapore, Singapore; 6 Department of Ophthalmology, Yong Loo Lin School of Medicine, National University of Singapore, Singapore, Singapore; 7 Centre for Quantitative Medicine, Duke-NUS Graduate Medical School, Singapore, Singapore; 8 Department of Ophthalmology, National University Hospital, Singapore, Singapore; 9 Department of Medicine, Yong Loo Lin School of Medicine, National University of Singapore, National University Health System, Singapore, Singapore; 10 Department of Epidemiology, Graduate School of Public Health; and University of Pittsburgh Cancer Institute, University of Pittsburgh, Pittsburgh, Pennsylvania USA; 11 Duke-NUS Graduate Medical School Singapore, Singapore, Singapore; 12 Yong Loo Lin School of Medicine, National University of Singapore, Singapore, Singapore; 13 National University Heart Centre, National University Health System, Singapore, Singapore; 14 School of Public Health and Community Medicine, Hebrew University of Jerusalem, Jerusalem, Israel

## Abstract

Recent genome-wide association studies (GWAS) have identified multiple loci associated with coronary artery disease (CAD) among predominantly Europeans. However, their relevance to multi-ethnic populations from Southeast Asia is largely unknown. We performed a meta-analysis of four GWAS comprising three Chinese studies and one Malay study (Total N = 2,169 CAD cases and 7,376 controls). Top hits (*P* < 5 × 10^−8^) were further evaluated in 291 CAD cases and 1,848 controls of Asian Indians. Using all datasets, we validated recently identified loci associated with CAD. The involvement of known canonical pathways in CAD was tested by Ingenuity Pathway Analysis. We identified a missense SNP (rs2075291, G > T, G185C) in *APOA5* for CAD that reached robust genome-wide significance (*Meta P* = 7.09 × 10^−10^, OR = 1.636). Conditional probability analysis indicated that the association at rs2075291 was independent of previously reported index SNP rs964184 in *APOA5*. We further replicated 10 loci previously identified among predominantly Europeans (*P:* 1.33 × 10^−7^–0.047). Seven pathways (*P*: 1.10 × 10^−5^–0.019) were identified. We identified a missense SNP, rs2075291, in *APOA5* associated with CAD at a genome-wide significance level and provided new insights into pathways contributing to the susceptibility to CAD in the multi-ethnic populations from Southeast Asia.

## Introduction

Coronary artery disease (CAD) is a common heart disorder. In recent years, CAD has been a primary cause of death in most countries worldwide, including large Asian countries such as India and China^1^. Heritability of CAD is estimated to be 30% to 60%^2^. Thus far, genome wide association studies (GWAS) have identified 56 loci associated with CAD among populations of predominantly European ancestry^3^, explaining approximately 13% of genetic variation in CAD. Some index variants such as those at the cyclin-dependent kinase inhibitor 2A/cyclin-dependent kinase inhibitor 2B (*CDKN2A/CDKN2B*) gene on 9p21 have been validated to confer CAD risk in multiple ethnic groups^4–7^. However, the *ADTRP-C6orf105* locus detected in Chinese has not been confirmed among other populations, such as the Europeans and South Asians^3,8^. Such discrepancies could be due to differences in linkage disequilibrium (LD) patterns and in allele frequencies between populations, or due to true ethnic-specific genetic effects. Therefore, replication efforts for known CAD associated loci, particularly those reported very recently^3^, are needed to corroborate initial findings and determine the transferability of identified loci among multiple ethnic groups.

Singapore is a multiethnic, multicultural society comprising mainly Chinese, Malays and Asian Indians. Previous CAD GWAS conducted on subjects of Chinese ancestry have mainly drawn samples from the Northern provinces in China^3,7,9^. In Singapore however, the Chinese are predominantly descendants of immigrants from the Southern provinces and may be genetically distinct due to the existence of a north–south genetic cline^10^. It is noteworthy that there is a lack of CAD GWAS of Southern Chinese, Malays and Asian Indians. Therefore, additional genetic studies in these major Asian populations may identify additional susceptibility loci, and determine if common genetic variants that were identified from populations of predominantly European ancestry are transferrable to the multi-ethnic populations from Southeast Asia. In this study, we aim to (i) discover novel loci associated with CAD and (ii) evaluate the transferability of known CAD loci among Southern Chinese, Malays and Asian Indian populations.

## Results

### A missense SNP in the *APOA5* (Apolipoprotein A5) gene, rs2075291, was found to be associated with CAD at a level of genome-wide significance in multi-ethnic cohorts from Southeast Asia comprising subjects of Chinese, Malay and Asian Indian ancestry

**Figure 1:**
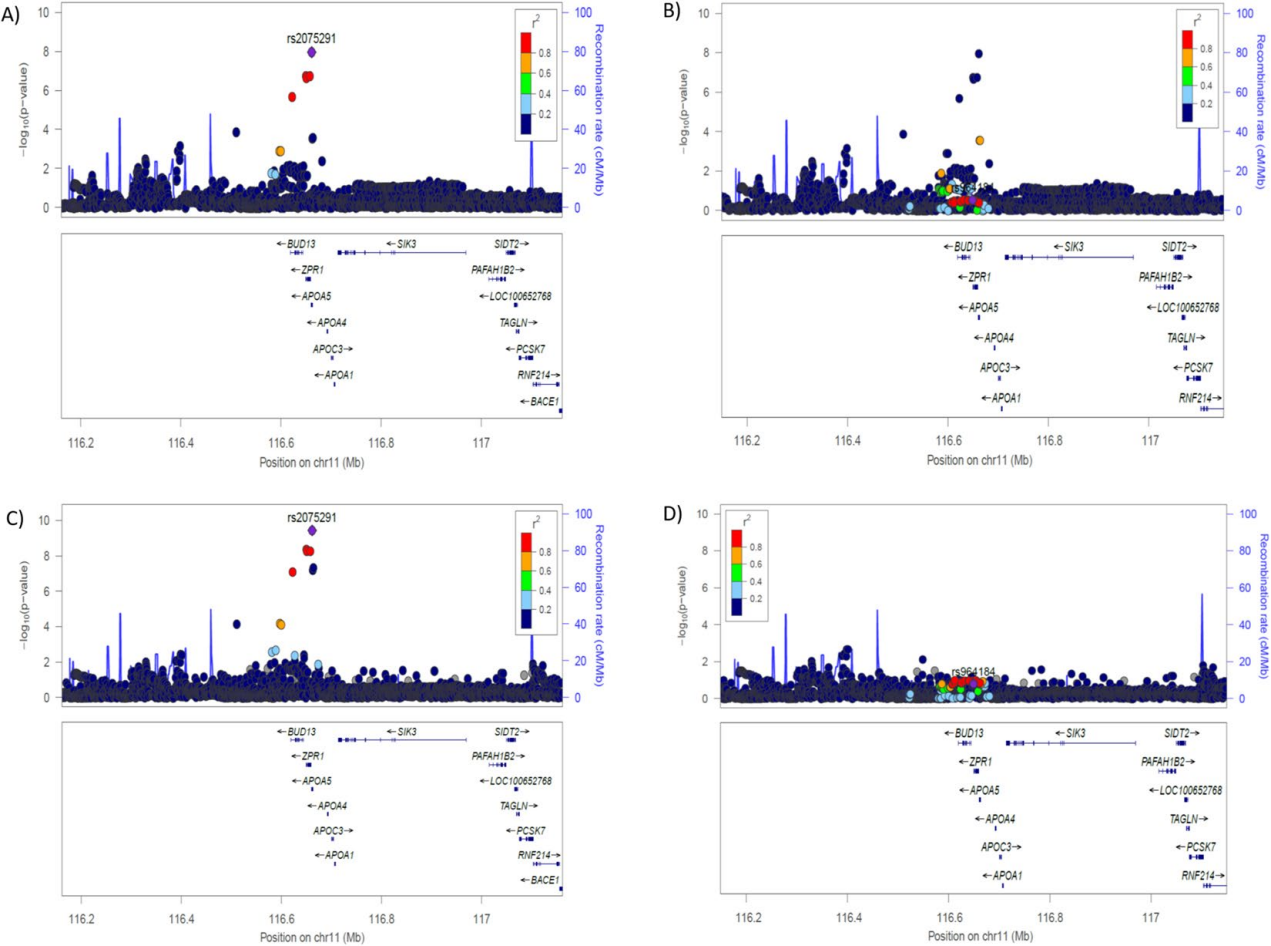
Regional SNP association plots at gene locus containing rs2075291 and rs964184 in the discovery Chinese and Malay Meta-analysis. Gene annotations from the RefSeq track of the UCSC Gene Browser (LocusZoom http://csg.sph.umich.edu/locuszoom/). (**A**) Association levels with rs2075291 as index SNP. (**B**) Association level for rs964184 as index SNP. (**C**) Regional association levels of SNPs after adjustments for rs964184 genotypes. (**D**) Regional association levels of SNPs after adjustments for rs2075291 genotypes. Index SNP of plots indicated by purple diamonds.

QQ and Manhattan plots from discovery meta-analysis of Chinese and Malay CAD case-control datasets [(1) Singapore Chinese Health Study CAD cases and controls (SCHS), (2) Singapore Coronary Artery Disease Genetics Study (SCADGENS) Chinese CAD cases and controls from Singapore Prospective Study Programme (SP2), (3) SCADGENS Chinese cases and controls from Singapore Chinese Eye Study (SCES) and (4) SCADGENS Malay cases and controls from Singapore Malay Eye Study (SiMES)] are provided in Supplementary Figures I and II. The rs2075291 SNP (c.553 G > T, p.G185C, chr11:116661392, Build 37) reached the threshold of genome-wide significance in the Chinese and Malay populations (*Meta P* = 1.10 × 10^−8^, OR = 1.587, Fig. 1A and Table 1). Supplementary Table I provides details of all SNPs with Meta *P* < 10^−5^ in the discovery meta-analysis of Chinese and Malay CAD case-control datasets. This SNP was further replicated (*P* = 1.02 × 10^−3^, OR = 5.197, Table 1) in the Asian Indian study [SCADGENS Asian Indian CAD cases and controls from Singapore Indian Eye Study (SINDI)], and meta-analysis of all datasets showed robust associations (*Meta P* = 7.09 × 10^−10^, OR = 1.636, Table 1), with minimal between-study heterogeneity (*P*_het = 0.161). The base pair change of rs2075291 (G > T) causes a substitution of a cysteine for a glycine residue at amino acid 185 (G185C). Rs2075291 is rare (MAF < 1%) in the Europeans^11^ and has a MAF of approximately 7% in the Chinese, 4.6% in the Malays and 1.4% in the Asian Indians from our datasets. Conditional probability analysis indicated that the association at rs2075291 was independent of previously reported index SNPs, rs964184 (*P*-value = 3.69 × 10^−10^, OR = 1.659, Fig. 1C and Table 2) and rs662799 (−1131C > T promoter polymorphism) in *APOA5* (*P*-value = 5.70 × 10^−7^, OR = 1.539, Table 2).

**Table 1:** Association results for rs2075291 in individual datasets and after meta-analysis procedures.

	SCHS	SCADGENS/SCES	SCADGENS/SP2	SCADGENS/SiMES	Meta SG East-Asians	SCADGENS/SINDI	Meta SG ALL
N	718 + 1,262	631 + 1,713	429 + 2,189	391 + 2,212	2,169 + 7,376	291 + 1,848	2,460 + 9,223
SNP	rs2075291	rs2075291	rs2075291	rs2075291	rs2075291	rs2075291	rs2075291
Chr	11	11	11	11	11	11	11
Pos (Build 37)	116661392	116661392	116661392	116661392	116661392	116661392	116661392
EA	A	A	A	A	A	A	A
EAF	0.064	0.076	0.071	0.046	0.064	0.014	0.055
P	7.60 × 10^−4^	3.41 × 10^−3^	0.028	2.65 × 10^−3^	**1.10** × **10** ^**−8**^	1.02 × 10^−3^	**7.09** × **10** ^**−10**^
OR	1.594	1.537	1.459	1.937	1.587	5.197	1.636
95% CI	1.342–1.864	1.249–1.825	1.122–1.796	1.506–2.368	1.428–1.746	4.213–6.181	1.479–1.793
P_het	NA	NA	NA	NA	0.776	NA	0.161

EA: Effect allele; EAF: Effect allele frequency; OR: odds ratio; CI: confidence interval; P_het: P values for Cochran’s Q.

**Table 2:** Conditional probability analysis for *APOA5* variants (rs2075291 and rs964184) with CAD in individual datasets used in the study and after meta-analysis.

rsid	Chr	Pos (Build 37)	EA	SCHS		SCADGENS/SCES		SCADGENS/SP2		SCADGENS/SiMES		SCADGENS/SINDI		META		
				P	OR	P	OR	P	OR	P	OR	P	OR	OR	95% CI	P
rs2075291 association conditioned on rs964184 genotypes	11	116661392	A	0.002	1.547	0.002	1.578	0.016	1.530	0.002	2.022	0.001	5.008	1.659	1.500–1.818	**3.69** × **10** ^**−10**^
rs964184 association conditioned on rs2075291 genotypes	11	116648917	C	0.287	1.093	0.146	0.877	0.027	0.798	0.197	0.884	0.573	1.066	0.941	0.857–1.025	0.154
rs2075291 association conditioned on rs662799 genotypes	11	116661392	A	4.32 × 10^−4^	1.710	0.046	1.378	0.295	1.214	0.018	1.698	0.001	4.966	1.539	1.300–1.822	**5.70 × 10** ^**−7**^
rs662799 association conditioned on rs2075291 genotypes	11	120000000	A	0.199	1.112	0.153	0.877	0.043	0.808	0.187	0.877	0.976	1.004	0.940	0.863–1.025	0.160

EA: Effect allele; OR: odds ratio; CI: confidence interval.

Similar to previous reports from candidate gene studies^12–18^, the *A* allele of rs2075291 showed strong associations with HDL-C levels (Beta = −0.401, *P* = 9.76 × 10^−12^, Supplementary Table II) and fasting TG levels (Beta = 0.392, *P* = 9.80 × 10^−11^, Supplementary Table II) in our SP2 dataset and modest association with HDL-C (Beta = −0.196, *P* = 0.002, Supplementary Table II) and non-fasting levels of TG (Beta = 0.226, *P* = 0.001, Supplementary Table II) in the SCHS dataset. Blood lipids were associated with CAD in the SCHS dataset (*P* < 0.002, Supplementary Table III). To determine if the identified CAD effects of rs2075291 were mediated through blood lipids we adjusted the observed CAD association at rs2075291 in the SCHS case-control dataset for LDL-C, HDL-C and TG levels. Inclusion of the individual lipid levels in the regression model did not diminish the significant effect of rs2075291 and CAD in the SCHS study (OR = 1.580, 95% CI = 1.194–2.090, *P* = 0.001, Supplementary Table IV). The association of rs2075291 with CAD in the SCHS was also not diminished with further adjustments for additional CAD risk factors (BMI, blood pressure and HbA1c) in the SCHS case-control study (OR = 1.581, 95% CI = 1.140–2.194, *P* = 0.006, Supplementary Table IV).

The G > T substitutions at rs2075291 is predicted to be probably damaging with a score of 0.997 by PolyPhen-2^19^, and by PROVEAN^20^ (PROVEAN score = −3.44), suggesting a deleterious effect of the substitution on protein function.

### Transferability of previously identified CAD index loci in the Chinese, Malay and Asian Indian populations

We validated CAD associations of index SNPs using meta-analysis of data from all Chinese, Malay and Asian Indian datasets. Out of the 56 independent index SNPs from CAD associated loci discovered from recent GWAS studies^3^ in primarily European ancestry, we evaluated 46 independent index SNPs (r^2^ < 0.2) that were genotyped or imputed and passed GWAS QC thresholds in all the multi-ethnic datasets (Table 3). Among the 46 known index SNPs, rs4977574 from *CDKN2A/2B* locus, rs9349379 from *PHACTR1* locus, rs7173743 from *ADAMTS7* locus were significantly replicated in our study (adjusted *Meta P* = 0.014–6.12 × 10^−6^, Table 3). Additionally 7 index SNPs near or at *ABO, PDGFD, TCF21, ADTRP/C6orf105, APOE/APOC1, PMAIP1/MC4R* and *LPL* loci showed suggestive associations in our study (*Meta P* ranging from 0.003–0.047, Table 3). A binomial-test revealed significant enrichment of association signals (Binomial *P* = 6.28 × 10^−5^) at these index SNPs from our data. At 42 of the 46 known index SNPs, the directions of the genetic effect in our meta-analysis were also consistent with those first observed in studies with subjects of predominantly European ancestry (Binomial *P* < 1 × 10^−6^, Table 3). For all replicating SNPs, except rs12413409 at *CYP17A1/CNNM2/NT5C2*, minimal between-study heterogeneity was observed (P_het > 0.07, Table 3) in the meta-analysis. The association of rs12413409 was recalculated in random effects meta-analysis and the significance level weakened (*P* = 0.255, OR = 0.898, 95% CI = 0.745–1.081). The GRS of all 46 index SNPs showed a strong association with CAD (*P* = 5.51 × 10^−16^, Table 3).

**Table 3:** Association results of previously reported index SNPs among predominantly Europeans after meta-analysis of the 5 Singaporean Asian datasets (2,460 cases; 9,223 controls).

rsid	Locus	Chr	Pos (Build 37)	EA (EAF)**	OR (95% CI)**	EA	EAF	P_META	P_META_adj	OR (95% CI)	P_het	Power
rs4977574	*9p21*	9	22098574	G (0.49)	1.21 (1.19, 1.23)	G	0.48	1.33 × 10^−7^	**6.12** × 10^−**6**^	1.20 (1.12, 1.29)	0.765	99.99%
rs9349379	*PHACTR1*	6	12903957	G (0.43)	1.14 (1.12, 1.16)	G	0.70	1.97 × 10^−4^	**9.04** × 10^−**3**^	1.15 (1.07, 1.23)	0.493	98.07%
rs7173743	*ADAMTS7*	15	79141784	T (0.56)	1.08 (1.06, 1.10)	C	0.50	2.95 × 10^−4^	**1.36** × 10^−**2**^	0.88 (0.82, 0.94)	0.355	69.92%
rs532436	*ABO*	9	136149830	A (0.19)	1.12 (1.09, 1.14)	A	0.18	2.51 × 10^−3^	0.115	1.15 (1.05, 1.26)	0.787	79.10%
rs974819	*PDGFD*	11	103660567	T (0.33)	1.07 (1.04, 1.09)	C	0.37	2.52 × 10^−3^	0.116	0.90 (0.84, 0.96)	0.088	58.20%
rs12202017	*TCF21*	6	134173151	A (0.70)	1.07 (1.05, 1.09)	G	0.52	3.08 × 10^−3^	0.142	0.90 (0.84, 0.97)	0.536	58.83%
rs6903956	*ADTRP-C6orf105*	6	11774583	A (0.35)	1.00 (0.98, 1.02)	G	0.95	5.24 × 10^−3^	0.241	0.82 (0.72, 0.94)	0.072	5.00%^‡^
rs12413409	*CYP17A1-CNNM2-NT5C2*	10	104719096	G (0.89)	1.08 (1.05, 1.11)	A	0.27	2.12 × 10^−2^	0.974^†^	0.91 (0.84, 0.99)^†^	**2.97 × **10^−**4**^	56.26%
rs2075650	*APOE-APOC1*	19	45395619	G (0.13)	1.07 (1.04, 1.11)	G	0.08	2.31 × 10^−2^	1.000	1.14 (1.02, 1.29)	0.963	26.00%
rs663129	*PMAIP1-MC4R*	18	57838401	A (0.26)	1.06 (1.04, 1.08)	A	0.16	2.69 × 10^−2^	1.000	1.11 (1.01, 1.21)	0.103	32.09%
rs17411031	*LPL*	8	19852310	C (0.74)	1.05 (1.03, 1.08)	G	0.21	4.73 × 10^−2^	1.000	0.92 (0.84, 1.00)	0.268	25.45%
rs646776	*SORT1*	1	109818530	T (0.75)	1.11 (1.08, 1.13)	T	0.94	0.065	1.000	1.12 (0.99, 1.26)	0.291	51.79%
rs8042271	*MFGE8-ABHD2*	15	89574218	G (0.90)	1.10 (1.06, 1.14)	A	0.28	0.066	1.000	0.93 (0.85, 1.01)	0.472	75.00%
rs17465637	*MIA3*	1	222823529	C (0.66)	1.08 (1.06, 1.10)	C	0.63	0.077	1.000	1.07 (0.99, 1.15)	0.510	67.30%
rs7212798	*BCAS3*	17	59013488	C (0.15)	1.08 (1.05, 1.11)	C	0.14	0.095	1.000	1.09 (0.98, 1.22)	0.407	36.47%
rs7692387	*GUCY1A3*	4	156635309	G (0.81)	1.07 (1.05, 1.10)	A	0.20	0.099	1.000	0.93 (0.85, 1.01)	0.282	40.09%
rs10953541	*7q22*	7	107244545	C (0.78)	1.05 (1.03, 1.08)	T	0.15	0.114	1.000	0.92 (0.84, 1.02)	0.972	19.44%
rs9970807	*PPAP2B*	1	56965664	C (0.92)	1.13 (1.10, 1.17)	T	0.03	0.125	1.000	0.87 (0.73, 1.04)	0.202	35.73%
rs16986953	*AK097927*	2	19942473	A (0.10)	1.09 (1.06, 1.12)	A	0.30	0.134	1.000	1.06 (0.98, 1.15)	0.422	73.70%
rs2048327	*SLC22A3-LPAL2-LPA*	6	160863532	C (0.35)	1.06 (1.04, 1.08)	C	0.43	0.136	1.000	1.05 (0.98, 1.13)	0.568	46.03%
rs1561198	*VAMP5-VAMP8-GGCX*	2	85809989	T (0.46)	1.06 (1.04, 1.08)	T	0.39	0.137	1.000	1.05 (0.98, 1.13)	0.378	45.04%
rs72743461	*SMAD3*	15	67441750	C (0.80)	1.07 (1.05, 1.1)	A	0.03	0.157	1.000	0.89 (0.75, 1.05)	0.810	16.19%
rs4593108	*EDNRA*	4	148281001	C (0.80)	1.07 (1.05, 1.10)	G	0.34	0.159	1.000	0.95 (0.89, 1.02)	0.822	56.55%
rs2107595	*HDAC9*	7	19049388	A (0.20)	1.08 (1.05, 1.10)	A	0.33	0.164	1.000	1.05 (0.98, 1.13)	0.613	65.63%
rs501120	*CXCL12*	10	44753867	T (0.81)	1.08 (1.06, 1.11)	C	0.34	0.191	1.000	0.95 (0.88, 1.02)	0.320	63.09%
rs56336142	*KCNK5*	6	39134099	T (0.81)	1.07 (1.04, 1.09)	C	0.14	0.194	1.000	0.94 (0.85, 1.03)	0.688	33.37%
rs6725887	*WDR12*	2	203745885	C (0.11)	1.14 (1.11, 1.18)	C	0.02	0.211	1.000	1.18 (0.91, 1.52)	0.277	21.58%
rs9319428	*FLT1*	13	28973621	A (0.31)	1.04 (1.02, 1.06)	A	0.46	0.212	1.000	1.05 (0.97, 1.14)	0.340	24.16%
rs2681472	*ATP2B1*	12	90008959	G (0.20)	1.08 (1.05, 1.10)	G	0.31	0.289	1.000	1.04 (0.97, 1.12)	0.048	62.66%
rs17087335	*REST - NOA1*	4	57838583	T (0.21)	1.06 (1.04, 1.09)	T	0.39	0.299	1.000	1.04 (0.97, 1.12)	0.376	44.41%
rs180803	*POM121L9P-ADORA2A*	22	24658858	G (0.97)	1.20 (1.13, 1.27)	T	0.08	0.306	1.000	0.94 (0.82, 1.06)	0.601	85.78%
rs216172	*SMG6*	17	2126504	C (0.35)	1.05 (1.03, 1.07)	C	0.28	0.346	1.000	1.04 (0.96, 1.12)	0.027	30.61%
rs55940034	*COL4A1/A2*	13	111043309	G (0.27)	1.07 (1.04, 1.09)	G	0.11	0.362	1.000	1.05 (0.94, 1.17)	0.218	31.09%
rs11830157	*KSR2*	12	118265441	G (0.36)	1.04 (1.02, 1.06)	G	0.25	0.444	1.000	0.97 (0.90, 1.05)*	0.826	20.37%
rs964184	*ZNF259-APOA5-APOA1*	11	116648917	G (0.18)	1.05 (1.03, 1.08)	C	0.79	0.477	1.000	0.97 (0.89, 1.05)	0.088	26.22%
rs11556924	*ZC3HC1*	7	129663496	C (0.69)	1.08 (1.05, 1.10)	T	0.06	0.486	1.000	0.95 (0.81, 1.10)	0.728	26.30%
rs2252641	*ZEB2-ACO74093.1*	2	145801461	C (0.48)	1.03 (1.01, 1.05)	C	0.79	0.550	1.000	1.03 (0.94, 1.11)	**0.043**	12.73%
rs2954029	*TRIB1*	8	126490972	A (0.55)	1.04 (1.03, 1.06)	T	0.52	0.583	1.000	0.98 (0.92, 1.05)	0.988	24.44%
rs46522	*UBE2Z*	17	46988597	T (0.51)	1.04 (1.02, 1.06)	T	0.69	0.605	1.000	1.02 (0.95, 1.10)	0.796	22.83%
rs4845625	*IL6R*	1	154422067	T (0.45)	1.05 (1.03, 1.07)	C	0.48	0.649	1.000	0.98 (0.92, 1.05)	**0.048**	34.39%
rs273909	*SLC22A4-SLC22A5*	5	131667353	G (0.12)	1.06 (1.03, 1.09)	G	0.03	0.704	1.000	0.97 (0.84, 1.13)*	0.951	16.92%
rs7214245	*RAI1-PEMT-RASD1*	17	17591759	T (0.56)	1.04 (1.02, 1.06)	A	0.08	0.727	1.000	0.98 (0.87, 1.10)	0.340	12.61%
rs17514846	*FURIN-FES*	15	91416550	A (0.44)	1.05 (1.03, 1.07)	A	0.16	0.730	1.000	1.02 (0.93, 1.11)	0.265	25.11%
rs2895811	*HHIPL1*	14	100133942	C (0.41)	1.04 (1.02, 1.06)	C	0.24	0.789	1.000	1.01 (0.93, 1.09)	0.347	20.82%
rs515135	*APOB*	2	21286057	C (0.79)	1.07 (1.04, 1.10)	C	0.90	0.973	1.000	1.00 (0.88, 1.14)*	0.383	23.18%
rs11206510	*PCSK9*	1	55496039	T (0.85)	1.08 (1.05, 1.11)	C	0.05	0.974	1.000	1.00 (0.85, 1.18)*	0.860	17.39%
GRS	—	—	—	—	—	—	—	5.51 × 10^−16^	—	1.05 (1.04, 1.06)	0.591	—

EA: Effect allele; EAF: Effect allele frequency; OR: odds ratio; CI: confidence interval; MA: minor allele; P_META_adj: P_META adjusted for 46 tests; P_het: P values for Cochran’s Q; GRS: genetic risk score. *Inconsistent direction with previous GWAS. **EF and OR from the reference: CARDIoGRAMplusC4D Consortium. A comprehensive 1000 Genomes-based genome-wide association meta-analysis of coronary artery disease. Nat Genet. 2015: 47: 1121–1130. ^†^Shown here in fixed effect, and recalculated in random effect with P-meta = 0.255, OR = 0.898. ^‡^Shown here the power calculation based on the ref.^3^, and recalculated based on the ref.^9^, Power = 99.99%.

We next tested for involvement of known canonical pathways using 49 genes within an LD block containing (r^2^ > 0.2 in 1000 G ASN populations, Supplementary Table V) rs2075291 and 11 validated index SNPs showing significant and suggestive associations in our study. Seven pathways that were significantly implicated included nuclear receptor activations (“LXR/RXR activation” and “FXR/RXR activations”), “Atherosclerosis Signaling”, lipid transport (“Clathirin mediated Endocytosis Signaling”), immune cell responses (“IL-12 Signaling and Production in Macrophages”, “Production of Nitric Oxide and Reactive Oxygen Species in Macrophages) and “Glioma Signaling” (*P*-value 1.10 × 10^−5^–0.019, Supplementary Table VI and Supplementary Figure III). These multiple pathways involve the genes *APOE, APOA4, APOC4, LPL, APOC1, APOA5, PDGFD, CDKN2A* and *CDKN2B*. In FUMAgwas analysis, the most significant pathways were lipid transport, metabolism and digestion mobilization, which involve *APOA5, APOE* and *LPL* (*P*-value 1.10 × 10^−5^–5.00 × 10^−4^, Supplementary Figure IV). DEPICT gene set enrichment included multiple functional classes relevant to lipid metabolism, ie, sphingolipid metabolism, glycosphingolipid metabolism, positive regulation of steroid metabolic process and glycosphingolipid biosynthesis. In addition, there were multiple gene sets associated with abnormal blood vessel function, including regulation of blood vessel size, vascular process in circulatory system and increased vasodilation (Supplementary Table VII).

We further evaluated associations of regional SNPs in LD (r^2^ > 0.2 in 1000 G ASN populations) with the previously reported index SNPs that did not significantly replicate in our datasets. At one locus, rs12202017 (*TCF21* gene), we identified a regional SNP with a significant association in the Chinese, Malay and Asian Indian datasets (rs9375986, adjusted *meta P* = 0.019, Supplementary Table VIII and Supplementary Figure V). We further detected stronger and nominal associations (*Meta P:* 1.55 × 10^−3^–4.99 × 10^−2^) at 14 additional loci for regional SNPs that were in at least moderate LD (r^2^ > 0.2 in 1000 G ASN populations) with previously reported index SNPs that did not replicate (Supplementary Table VIII and Supplementary Figure VI).

## Discussion

In this study, we identified a missense SNP in *APOA5*, rs2075291, which is associated with CAD at a level of genome-wide significance. Previous candidate gene studies have investigated the association of variants at the *APOA5* locus, including rs2075291, with CAD, and findings were inconsistent across different populations^11,13,15–17,21–23^. Our study confirms this association using a GWAS approach in multi-ethnic populations from Southeast Asia.

Rs2075291 has a risk allele (*A* allele) frequency of approximately 7.0% in our Chinese population, 4.6% in the Malays and 1.4% in the Asian Indians. This variant is rare (MAF < 1%) in populations of European ancestry^11^. It is therefore likely that this variant may have been missed even by large-scale CAD GWAS evaluating populations of predominantly European origin^3,8^. However, this SNP was previously reported to be nominally associated with early onset MI in populations of European ancestry (8 mutation carriers in 6,721 cases and 2 mutation carriers in 6,711 controls, OR = 4.00, P = 0.109)^11^. It is noteworthy that this SNP was not identified by previous CAD/MI GWAS conducted in the Chinese^7,9^, Japanese^24–27^ and Korean populations^28^. It is likely that this SNP was not imputed by older HapMap panels or may have not been reported due to a weaker significance level. For example, data from a Chinese exome-wide association study for lipid levels further tested the association of the identified variants with CAD and showed similar risk of the *A* allele of rs2075291 for CAD (OR = 1.14, *P* = 9.60 × 10^−3^)^29^. Our results also highlighted the value of further large-scale genetic studies in additional ethnic groups that may uncover disease variants with ethnic-specific effects. The rs2075291 variant identified in our Southeast Asian study confers about 60% increased risk of CAD per copy of risk allele. The lower MAF and smaller sample size in the Asian Indians, may have accounted for the inflated effect estimate in the replication dataset. It is noteworthy that rs2075291 has a greater effect size than the well-replicated *CDKN2A/2B* locus. The *CDKN2A/2B* locus has the greatest single locus effect in large-scale GWAS comprising subjects predominantly of European ancestry and is associated with about 20% increased risk of CAD per copy of risk allele in our data and in the European populations.

We further showed that the rs2075291 association was independent of the reported GWAS index SNP, rs964184, at the *APOA5* locus^3^ as well as −1131C > T (rs662799) promoter polymorphism and a 3′ untranslated region polymorphism (rs2266788), which are strongly associated with CAD risk^30,31^. Adjustment of the observed signal for CAD at the *APOA5* locus with rs2075291 genotypes abolished all significant associations indicating that the rs2075291 is likely to be the lead SNP in the South-East Asian samples tested.

The human *APOA5* gene has four exons and three introns. Its gene product is a 369 amino acid apoAV plasma protein that is a component of very low-density lipoprotein (VLDL) and high-density lipoprotein (HDL). ApoAV indirectly activates lipoprotein lipase (LPL)–mediated triacylglycerol lipolysis by promoting triacylglycerol-rich lipoprotein binding to glycosylphosphatidylinositol HDL- binding protein 1 (GPIHBP1) at the endothelial cell surface where LPL resides^32^. Studies in animals and humans indicate that apoAV is crucial in TG metabolism^33–37^ and residues 171–188 may play a role in affecting the binding of apoAV to lipid interfaces^38^. The missense rs2075291 SNP, which gives rise to a cysteine for a glycine substitution at position 185 results in aberrant disulfide bond formation, thereby abrogating its lipoprotein interaction capability with VLDL and HDL^39^. In line with this evidence, the G > T substitution at rs2075291 is also predicted to have a deleterious effect on apoAV functions by PolyPhen-2 and PROVEAN. Rs2075291 is associated with elevated TG levels^12–17,40^ and lower HDL-C levels^18^ in other Asian populations as well as in our datasets.

Whether the effect of rs2075291 on the increased risk of CAD is through predisposition to an atherogenic lipid profile or a yet to be identified apoAV function per se, will need further investigations. Previous studies have shown that TG-mediated pathways may be causally associated with CAD through Mendelian randomization^30,41^. The *APOA5* −1131C > T promoter polymorphism, which confers the TG raising effect, is associated with risk of CAD^30^. Do *et al*.^11^ surveyed how rare mutations associated with plasma lipid trait contribute to early-onset MI risk in >86,000 individuals. At *APOA5*, carriers of rare non-synonymous mutations had higher plasma TG and had a 2.2-fold increased risk for MI. Mutations in other genes that affect TG levels are also associated with CAD, for example, a common gain-of-function LPL variant, S447X, is associated with low TG level and lower MI risk^42^ and in another study, the aggregate of rare mutations in the gene encoding apolipoprotein C3 (*APOC3*) was associated with lower TG levels, and contribute to a reduced risk of CAD^43^. Despite the evidence supporting a causal role for triglyceride-mediated pathways in CAD, the adjustment of TG and other lipid profiles did not weaken the CAD association in our study. Consistent with our observations, a previous smaller scale study also noted that the increased CAD risk associated with rs2075291 may be independent of traditional risk factors, including TG and HDL-C^16^. The lipid-independent mechanism for the association of the *APOA5* variant with CAD needs further investigations with larger sample size since our study was limited by the fact that the subjects’ lipid profiles were only available in the SCHS dataset (682 cases/1209 controls).

With our GWAS meta-analysis data we also evaluated the transferability of 46 independent CAD associated index SNPs identified previously from GWAS of predominantly European populations to the multi-ethnic populations from Southeast Asia. Among the known index CAD SNPs, we detected an overrepresentation of positive association signals with rs4977574 from *CDKN2A/2B* locus, rs9349379 from *PHACTR1* locus and rs7173743 from *ADAMTS7* locus being significantly replicated, while 7 index SNPs near or at *ABO, PDGFD, TCF21, ADTRP/C6orf105*, *APOE/APOC1, PMAIP1/MC4R* and *LPL* loci showed suggestive associations in our study. There was a strong and significant correlation between the reported ORs from studies of Caucasians and the observed ORs from our study (Pearson’s R = 0.468, *P* = 0.001, Table 3), and our study also showed a strong association between a GRS of 46 index SNPs and CAD risk (Table 3), suggesting that a fraction of these associations might be shared even if some loci were not significantly replicated in the current study. It should be noted that 3 loci (*ADTRP-C6orf105, GUCY1A3, ATP2B1*) out of the 46 loci were first detected in Han Chinese^3,7,9^, however, they were not significantly replicated probably due to a low power (Table 3). It is also possible that the Chinese subjects in our study are predominantly descendants of immigrants from the Southern provinces and may be genetically distinct^10^ from Chinese subjects from the Northern provinces that were used in previous studies. The strongest association replicated in this study is the *CDKN2A/CDKN2B* gene locus on 9p21 (rs4977574), which has been shown to be most consistently associated with CAD in multiple ethnicities^4,7,27,44,45^. Relatively fewer groups had reported its association with myocardial infarction^5,46^ and early onset of heart disease^47^.

Genes at these replicated loci may have direct involvements with the pathogenesis of CAD. *CDKN2A* and *2B* gene products, p16^INK4a^, p14^ARF^ and p15^INK4b^, are known to modulate the development of CAD by controlling macrophage and smooth muscle cell proliferation and apoptosis^48^. The *PHACTR1* gene, which encodes the protein phosphatase 1 and actin regulator 1 (PHACTR1), has been reported to be a major determinant of stenosis in coronary arteries^49^. *In vivo* experimental validation demonstrated that ADAMTS7 is proatherogenic, perhaps through modulation of vascular cell migration and matrix in atherosclerotic lesions^50^. Overexpression of ADAMTS7 promotes migration of vascular smooth muscle cells *in vitro* and aggravates neointimal thickening after carotid artery injury *in vivo*, likely through degradation of cartilage oligomeric matrix protein^51^.

A number of index SNPs tested however, failed to reach statistical significance in our study. The disparities between the results from Southeast Asian and Caucasian studies might be due to ethnic-specific risk variants, genetic architecture, namely MAF and LD, differences in environmental factors and/or modifications of genetic effects as a result of environmental interactions and reduced statistical power. Generally we were able to replicate the SNPs that showed higher power to detect such associations in our study (mean power of replicating SNPs = 89.3%, Table 3), while SNPs that did not replicate had a lower power in our study (mean power of SNPs that failed to replicate = 38.37%, Table 3).

Using genes within an LD block containing rs2075291 and 11 validated index SNPs showing at least suggestive associations in our study, we evaluated for their enrichments in canonical pathways. We identified significant associations at seven pathways in our study with strong relevance to CAD. Interestingly, our pathway-analysis revealed potential interplay between genes at various loci that may be involved in in CAD pathogenesis. For example, *APOE, APOA4, APOC4, LPL, APOC1 and APOA5* were involved in nuclear receptor (liver X receptors and retinoid X receptors) activations, which serve as cholesterol sensors in regulating the expression of multiple genes implicated in the cholesterol efflux, transport, and excretion. These pathways have been identified as potential therapeutic targets in cardiovascular diseases^52^.

In conclusion, we have shown that some known index CAD loci, first identified in subjects of predominantly European ancestry, are transferrable to the Chinese, Malay and Asian Indian populations. Our GWAS of multi-ethnic populations from Southeast Asia identified a missense SNP in *APOA5*, rs2075291, which is associated with susceptibility to CAD.

## Methods

### Overview of study populations

We performed a discovery stage meta-analysis of four multi-ethnic genome-wide association studies comprising three Chinese studies (SCHS, SCADGENS/SCES, SCADGENS/SP2) and one Malay study (SCADGENS/SiMES) (Total N = 2,169 cases and 7,376 controls). Genome wide hits (*P* < 5.0 × 10^−8^) from discovery stage meta-analysis (Supplementary Table I) were further evaluated in 291 CAD cases and 1,848 controls of Asian Indian ethnicity (SCADGENS/SINDI).

### Singapore Chinese Health Study

The Singapore Chinese Health Study (SCHS) is a population-based prospective cohort which began in 1993–1998 and includes 63,257 Singaporean Chinese aged 45–74 years at baseline^53,54^. The study population constituted of two of the largest dialect groups of Chinese in Singapore, the Cantonese and the Hokkiens (both originate from southeastern provinces in China). Upon recruitment, subjects were interviewed by research staff following a questionnaire that included information on demographic and lifestyle factors and family and medical history^55^.

The first follow-up interviews for this cohort were conducted between 1999 to 2004 and blood specimens were collected from 32,543 subjects mostly between 2000 and 2005, shortly after they were contacted for the first follow-up interviews. We used data from a nested case-control study of CAD. Cases and controls were both without a history of stroke or CAD at the time of blood collection based on self-report and data from the national Hospital Discharge Database. Cases were participants who went on to develop incident non-fatal acute myocardial infarction (AMI) or fatal CAD occurring between the date of specimen collection and 31 December 2010, and they were identified through linkage with three databases. (1) The national Hospital Discharge Database records inpatient discharges from public hospitals in Singapore. Up to 31 December 2010, all AMI subjects (ICD-9: 410) within the cohort were selected as potential cases. A cardiologist used the criteria of the Multi-Ethnic Study of Atherosclerosis^56^ to review the medical records of the potential cases and only confirmed AMI cases were included. (2) The Singapore Myocardial Infarction Registry (SMIR) ascertain AMI cases on the basis of evaluation of medical history^57^ with standard procedures. (3) The Singapore Registry of Births and Deaths codes causes of death, and only participants who died from ischemic heart disease (ICD-9: 410-414) were selected as cases in the study. We identified 762 participants with incident AMI or CAD death.

In this study, for each CAD case, two controls that were alive and free of CAD at the time of diagnosis of AMI or death of ischemic heart disease were matched to the cases on date of recruitment (±1 year), date of birth (±2 years), sex, father’s dialect group and the date of blood specimen collection (±6 months).

In the current analysis, we used data from 718 CAD and 1,262 controls from the nested case-control study within SCHS cohort that had complete GWAS data that passed QC procedures. All SCHS participants provided written informed consent, and the study was approved by the Institutional Review Board of the National University of Singapore. All methods were performed in accordance with the relevant guidelines and regulations.

### Singapore Coronary Artery Disease Genetics Study

SCADGENS is an ongoing multi-ethnic study from June 2011 that is designed to assess the genetic determinants of CAD in Singapore. The cohort enrolls patients undergoing diagnostic coronary angiography at National University Heart Centre, Singapore and who have angiographically-proven coronary artery stenosis of at least 50% in one or more epicardial coronary arteries or their branches. The diagnosis of MI was ascertained through review of medical records in accordance with the Universal Definition of Myocardial Infarction^58^. Enrichment for a more severe CAD phenotype was performed by including only patients with stenosis in major epicardial arteries (left main, left anterior descending artery, circumflex artery and right coronary artery). A total of 1,060 Chinese cases, 391 Malay cases and 291 Asian Indian cases met these inclusion criteria and were available for this analysis.

At recruitment, a face-to-face interview was performed by a research nurse based on a standardized questionnaire that asked for information related to demographic, alcohol consumption, smoking status, physical activities and medical history (hypertension, diabetes mellitus and hyperlipidemia). Written informed consent was obtained from all participants, and National Health Group Domain Specific Review Boards (NHG DSRB) has approved this study. All methods were performed in accordance with the relevant guidelines and regulations.

### Singapore Prospective Study Programme

The Singapore Prospective Study (SP2) is a cross-sectional study of participants aged between 24 to 95 years from the three major ethnic groups in Singapore including Chinese, Malays and Asian Indians^59^. It began with the invitation of 10,445 subjects from 4 population-based, cross-sectional studies conducted from 1982 to 1998 in Singapore to participate in a repeat examination from 2004 to 2007. A total of 7,742 subjects were recruited, comprising 5,499 Chinese, 1,405 Malays and 1,138 Asian Indians. These participants were invited for health examination and collection of blood specimen shortly after the home visit. In summary, 74.1% of 7,742 subjects (N = 5,736) had the questionnaire completed and 49.4% (N = 3,824) attended the health examination.

Interviewer-administered questionnaires were conducted in this study. Information on demographic, lifestyle factors, and medical history (CAD, stroke, diabetes mellitus, hyperlipidemia and hypertension) were included in the questionnaire. Among the participants, 5,094 provided fasting blood samples (fasting for at least 10 hours). Approximately half of them were genotyped using Illumina genotyping arrays. In this study, a subset of 2,189 ethnic Chinese subjects who reported not having CAD or stroke were available as control subjects for SCADGENS. Informed consent was obtained from all subjects, and this study was approved by the respective IRBs of NUS and Singapore General Hospital. All methods were performed in accordance with the relevant guidelines and regulations.

### Singapore Epidemiology of Eye Disease Study

The Singapore Epidemiology of Eye Disease (SEED) studies are population-based cohort studies, designed to evaluate the prevalence, incidence and risk factors of major eye disorders. At baseline, SEED recruited adults aged 40 to 80 years, residing in the southern west of Singapore, a fair representation of the Singapore population according to the 2000 Singapore Census^60,61^. The SEED study included three major racial/ethnic groups: the Singapore Malay Eye Study (SiMES) commenced in 2004, and the Singapore Chinese Eye Study (SCES) and the Singapore Indian Eye Study (SINDI) which commenced in 2007^61^. The three studies were all conducted by the Singapore Eye Research Institute (SERI). The ethnicity of Malay, Chinese and Indian were defined by a criteria set from Singapore Census^62^, and indicated on the National Registration Identity Card. All subjects were selected based on an age-stratified random sampling strategy (10 year age group). The final participation rate for SiMES, SCES and SINDI were 3,280 (78.7% participant rate of 4,168 eligible participants), 3,400 (75.6% response rate of 4,497 eligible participants) and 3,300 (72.8% response rate of 4,533 eligible participants)^63^ with initial sampling frame 5,600, 6,350 and 6,350, respectively. All subjects underwent a standardized examination procedure and detailed questionnaires^60,61^, including lifestyle factors, medication information, surgical history and social economic status (housing status, marital status, education, occupation, etc.). In this study, a subset of 1,713 SCES subjects, 2,212 SiMES subjects and 1,848 SINDI subjects who reported not having CAD or stroke were available as control subjects for SCADGENS. All participants gave their written informed consents. The study followed the principles of the Declaration of Helsinki and was approved by the SERI IRB. The detailed methodology of the three studies has been previously published^60,61,64^. All methods were performed in accordance with the relevant guidelines and regulations.

### Genotyping and quality control

We performed a meta-analysis (2,169 CAD cases and 7,376 controls) of four multi-ethnic genome-wide association studies comprising three Chinese studies (SCHS, SCADGENS/SCES, SCADGENS/SP2) and a Malay study (SCADGENS/SiMES). The clinical characteristics of the 5 studies are shown in Supplementary Table III. We further tested for involvement of known canonical pathways for loci that had significant associations in our study using Ingenuity Pathway Analysis.

Samples from SCHS and SCADGENS were genotyped using IlluminaHumanOmniZhongHua-8 Bead Chip. Samples from SP2 were genotyped using HumanHap 610Quad, Illumina 1Mduov3 and Hap550 arrays^65^. Samples from SCES, SiMES and SINDI were genotyped using HumanHap 610Quad chip^65,66^. Based on sample quality control procedures described in Supplementary Table IX, we excluded duplicate samples (positive controls), samples with mismatched case-control status, samples with a low call rate (<98%), samples with excessive heterozygosity (outside the range of mean ± 3 standard deviation), samples with first and second-degree relatives (priortising the cases and/or samples with higher call-rates from each pair) and samples with discordant ethnicity from multi-ethnic populations from Southeast Asia. After sample quality control procedures, 718 cases and 1,262 controls from SCHS, 429 cases from SCADGENS and 2,189 controls from SP2,631 cases from SCADGENS and 1,713 controls controls from SCES, 391 cases from SCADGENS and 2,212 controls from SiMES, 291 cases from SCADGENS and 1,848 controls from SINDI were available for subsequent analysis (Supplementary Table III and IX).

Quality control procedures for genotyped SNPs excluded SNPs with low minor allele frequency (MAF < 0.01), non-single-nucleotide variant (non-SNV) SNPs, SNPs with significant departure from Hardy–Weinberg Equilibrium (HWE) in controls (*P* < 1 × 10^−5^) and SNPs with a low call rate (<95%, Supplementary Table X). Imputation procedures were performed using IMPUTE2^67^ and genotype calls were based on phase3 1000 G cosmopolitan panels. A total of 5,827,330 single-nucleotide polymorphisms (SNPs) from SCHS, 7,008,790 SNPs from SCADGENS and SP2, 7,230,115 SNPs from SCADGENS and SCES, 7,578,689 SNPs from SCADGENS and SiMES, and 7,436,598 SNPs from SCADGENS and SINDI were available for subsequent analyses after quality control procedures on imputed SNPs (Supplementary Table X). For imputed SNPs, SNP information scores were required to be 0.5 and monomorphic or rare SNPs (MAF < 0.01) were excluded. Detailed quality control procedures are available in Supplementary Table IX and X.

### Statistical analysis

Associations between SNPs and CAD phenotype were analyzed in an additive model with the adjustment for age, sex and population stratification (first three principal components). These analyses were performed with the genome association toolset, SNPTEST (version 2)^67^. Association of individual SNP genotype was quantified by the odds ratio (OR), 95% confidence interval (CI) and the association P for CAD.

Individual study results (SCHS, SCADGENS/SP2, SCADGENS/SCES and SCADGENS/SiMES) were subsequently pulled together (2,169 CAD cases and 7,376 controls) using the inverse variance-weighted meta-analysis, assuming a fixed effects model to derive overall pooled estimates and two-sided Ps using the META programme (METAv1.7). A Meta *P* < 5 × 10^−8^ was used to indicate genome-wide significance. Cochran’s Q was used to assess between-study heterogeneity and SNPs with Qpval <0.05 considered as significant. Study results showed little evidence of inflation (λ between 0.992 and 1.009, Supplementary Figure VII).

### Validation of known CAD associated index SNPs

Out of the 56 independent index SNPs from CAD associated loci discovered from recent GWAS studies^3^ in primarily European ancestry, we evaluated 46 independent index SNPs (r^2^ < 0.2) that were genotyped or imputed (using phase3 1000 G cosmopolitan panels) and passed GWAS QC thresholds in all the multi-ethnic datasets (Table 3). Of the 10 SNPs not available for analysis, one SNP had data only in three out of the five datasets, and the remaining 9 index SNPs failed QC procedures or had not been imputed or genotyped in all studies. Directional consistency and a Meta *P* < 1.09 × 10^−3^ (after Bonferonni correction for 46 tests) was used to indicate statistical significance and a Meta *P* < 0.05 was used to indicate suggestive/nominal significance. For SNPs that showed significant heterogeneity, meta-analysis procedures were repeated in the random effects model. The binomial test was used to test the enrichment of association signals at index SNPs from our data and the directional consistency of the genetic effect in our meta-analysis. Binomial *P* < 0.05 was used to indicate statistical significance. For index loci that did not show significant associations, we further evaluated the association of regional SNPs that were in at least moderate LD (r^2^ > 0.2) with the index SNP. Functional evaluation of SNPs were done using PolyPhen-2 (Polymorphism Phenotyping, v2)^19^ and PROVEAN (Protein Variation Effect Analyzer, v1.1.3)^20^.

We further tested the role of canonical pathways using all genes within an LD block containing (r^2^ > 0.2 in 1000 G ASN populations, Supplementary Table V) rs2075291 and 11 validated index SNPs that showed suggestive and significant associations in our study with Ingenuity Pathway Analysis (IPA) version24718999 and FUMAgwas^68^. Additional tissue enrichment and gene prioritization analysis were done by DEPICT (version 1.1)^69^ using rs2075291 and 11 validated index SNPs showing at least suggestive associations in our study.

All power calculations were carried out using QUANTO^70^, using previously reported effect estimates^3,9^ and observed MAF from the multi-ethnic cohorts (α = 5 × 10^−8^). We further tested regions that replicated among the multi-ethnic datasets for involvement in known pathways using Ingenuity Pathway Analysis (IPA) version 8.7 (Ingenuity® Systems, www.ingenuity.com). Weighted genetic risk score (GRS) using all 46 CAD associated SNPs was constructed. We multiplied the number of risk alleles at each CAD associated SNP by their reported effect estimates^3^. The weighted GRS were summed over all CAD associated SNPs, and divided by the average effect estimate of the 46 SNPs^3^. *P* < 0.05 (after Benjamini-Hochberg multiple testing correction) was used to indicate statistical significance.

### Data availability

All data generated or analyzed during this study are included in this published article (and its Supplementary Information files).

## Electronic supplementary material

Supplementary Information
