# Supplementary material for: Genome-wide association study identifies a missense variant at APOA5 for coronary artery disease in Multi-Ethnic Cohorts from Southeast Asia
Source: Sci Rep. 2017 Dec 20;7:17921. doi: 10.1038/s41598-017-18214-z (PMC5738399; doi:10.1038/s41598-017-18214-z)
Supplement: Supplementary file 1 — Supplementary Information [file 41598_2017_18214_MOESM1_ESM.doc]

## Supplementary Materials

**Title:**

Genome-wide association study identifies a missense variant at *APOA5* for coronary artery disease in Multi-Ethnic Cohorts from Southeast Asia

**Authors:**

Yi Han*, Rajkumar Dorajoo*, Xuling Chang, Ling Wang, Chiea-Chuen Khor, Xueling Sim, Ching-Yu Cheng, Yuan Shi, Yih Chung Tham, Wanting Zhao, Miao Ling Chee, Charumathi Sabanayagam, Miao Li Chee, Nicholas Tan, Tien Yin Wong, E-Shyong Tai, Jianjun Liu, Daniel Y.T. Goh, Jian-Min Yuan, Woon-Puay Koh, Rob M. van Dam, Adrian F. Low, Mark Yan-Yee Chan, Yechiel Friedlander†, Chew-Kiat Heng†

* Contributed equally

† Corresponding authors

## Supplementary Figures

**Supplementary Figure I:** Q-Q plot for SCHS, SCADGENS/SCES, SCADGENS/SP2 and SCADGENS/SiMES combined dataset (2,169 cases / 7,376 controls, λ = 1.026).


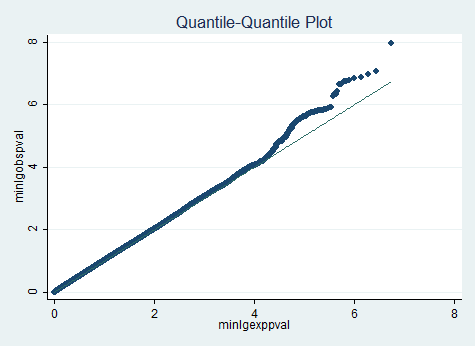


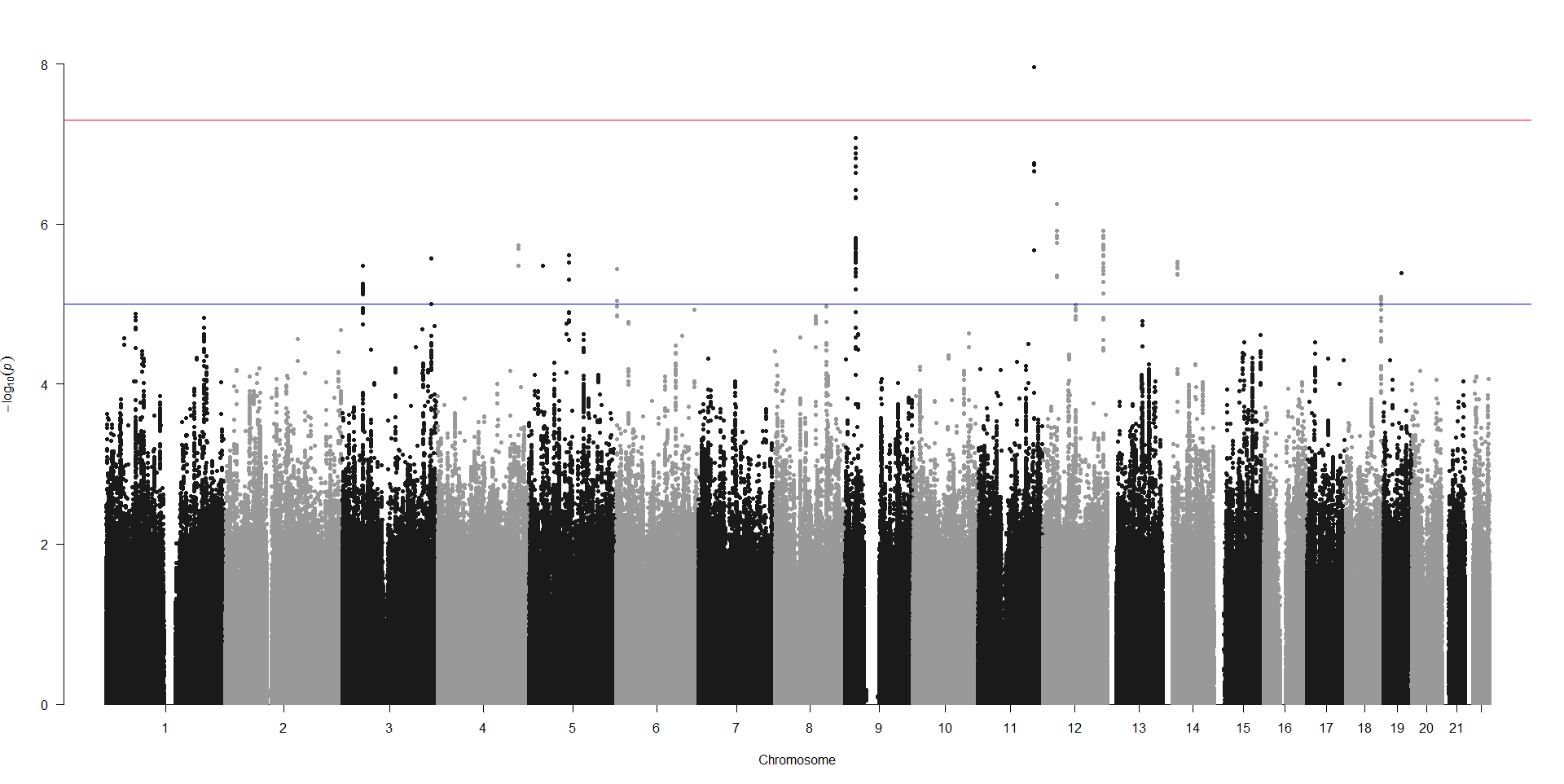
**Supplementary Figure II:** Manhattan plot for SCHS, SCADGENS/SCES, SCADGENS/SP2 and SCADGENS/SiMES combined dataset (2,169 cases / 7,376 controls).

**Supplementary Figure III:** Ingenuity Pathway Analysis (IPA) results of top canonical pathways identified with 49 genes for SNPs that are in at least moderate LD with rs2075291 and 11 SNPs that showed an association in our study (r^2^> 0.2 in 1000G ASN populations). The blue bars indicate Benjamini-Hochberg adjusted p-values for six significant pathways in the log scale (y-axis, left side). The dotted orange line indicates the ratio of the number of significant genes that mapped to a canonical pathway to the total number of genes involved in the pathway (y-axis, right side). Refer to supplementary table VI for list of genes that mapped to canonical pathways.

**
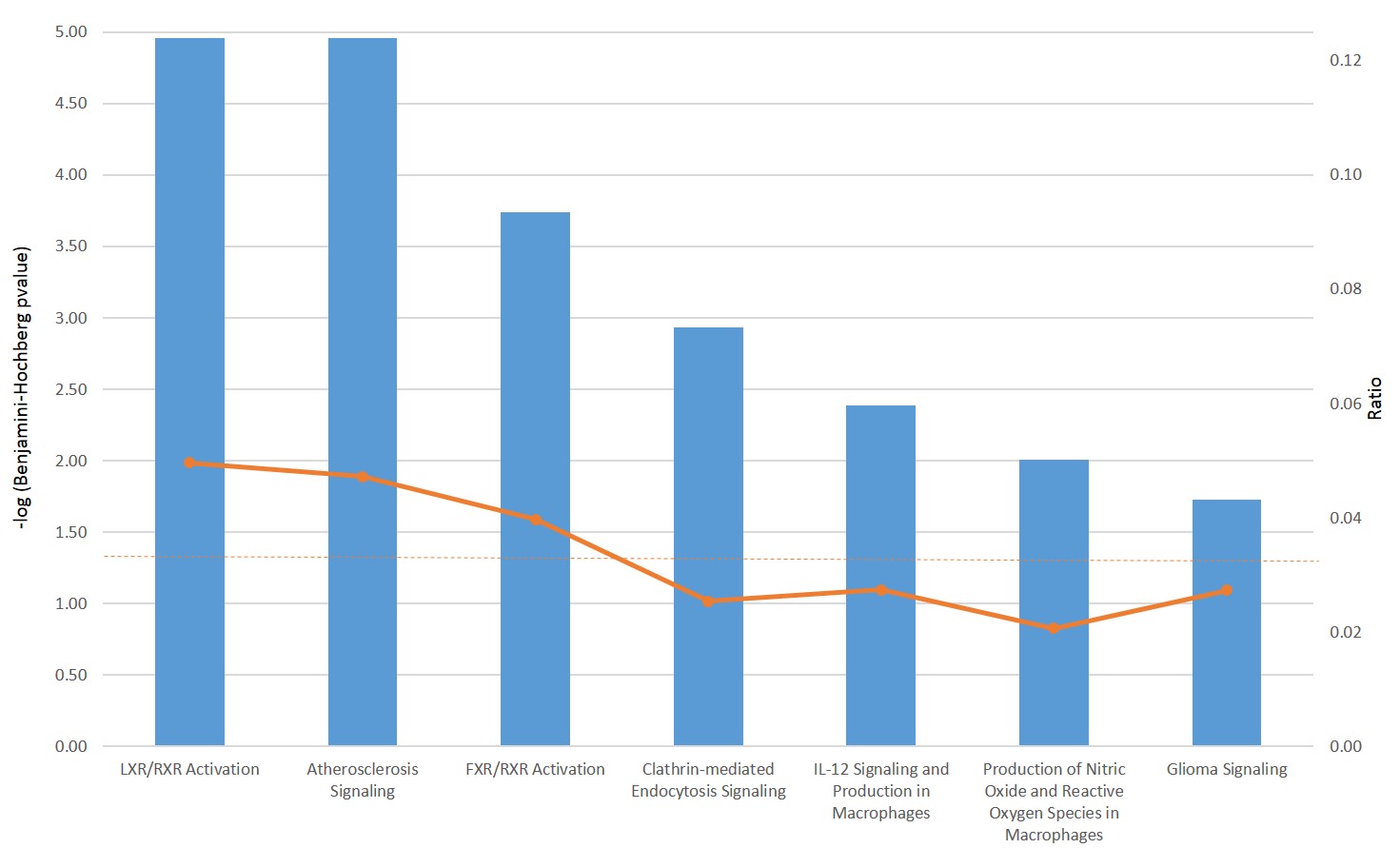
**

**Supplementary Figure IV:** FUMAgwas results of top pathway enrichment identified with 49 genes for SNPs that are in at least moderate LD
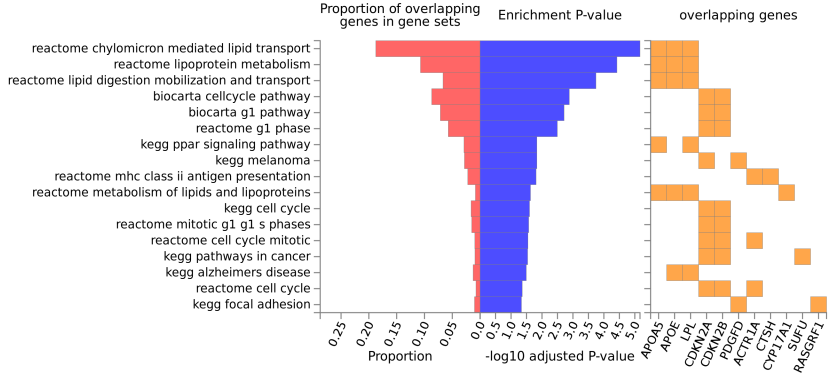
with rs2075291 and 11 SNPs that showed an association in our study (r^2^> 0.2 in 1000G ASN populations).

**Supplementary Figure V:** Regional SNP association plot of significant SNP (rs9375986 at chromosome 6) that was in at least moderate LD (r^2^ > 0.2 in 1000G ASN populations)with previously reported index SNP (rs12202017) that did not replicate, using CAD association p-values after GWAS meta-analysis. Rs9375986 is indicated by purple diamonds. (LocusZoom <http://csg.sph.umich.edu/locuszoom/>)

**Supplementary Figure VI:** Regional SNP association plots of 14 suggestive SNPs that were in at least moderate LD (r^2^ > 0.2 in 1000G ASN populations) with previously reported index SNPs that did not replicate, using CAD association p-values after GWAS meta-analysis. All plots plotted to include previously reported SNP (purple diamonds). A) rs2019090 at chromosome 11. B) rs2063192 at chromosome 6. C) rs11989309 at chromosome 8. D) rs12954782 at chromosome 18. E) rs7182288 at chromosome 15. F) rs75268817 chromosome 7. G) rs10008356 at chromosome 4. H) rs36113682 at chromosome 17. I) rs7368386 at chromosome 1. J) rs28566846 at chromosome 17. K) rs111485191 at chromosome 2. L) rs10017307 at chromosome 4. M) rs4970834 at chromosome 1. N) rs10189561 at chromosome 2. Index SNP of plots indicated by purple diamonds. Gene annotations from the RefSeq track of the UCSC Gene Browser (LocusZoom <http://csg.sph.umich.edu/locuszoom/>).

A)

B)

C)

D)

E)

F)

G)

H)

I)

J)

K)

L)

M)

N)

**Supplementary Figure VII:** Q-Q plot of 5 individual datasets. A) SCHS (718 cases / 1,262 controls), 5827330 SNPs, λ=0.992. B) SCADGENS+SCES (631 cases / 1,713 controls), 7230439 SNPs, λ=0.999. C) SCADGENS+SP2 (429 cases / 2,189 controls), 7008790 SNPs, λ=1.009. D) SCADGENS+SiMES (391 cases / 2,212 controls), 7378869 SNPs, λ=0.999. E) SCADGENS+SINDI (291 cases / 1,848 controls), 7437030 SNPs, λ=0.970.

D)

E)

A)


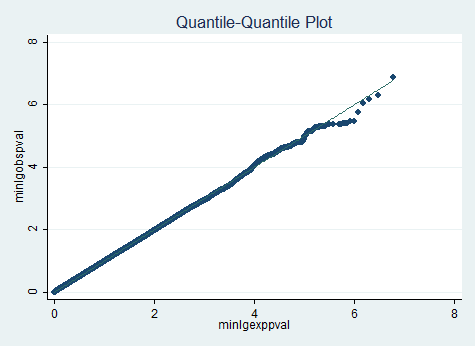


C)


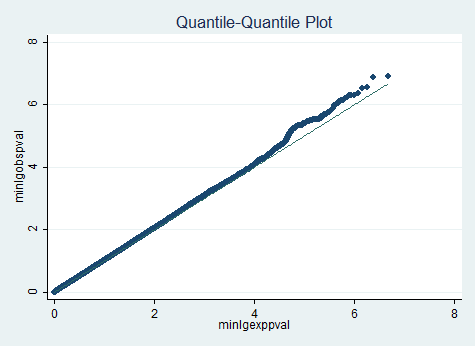


B)


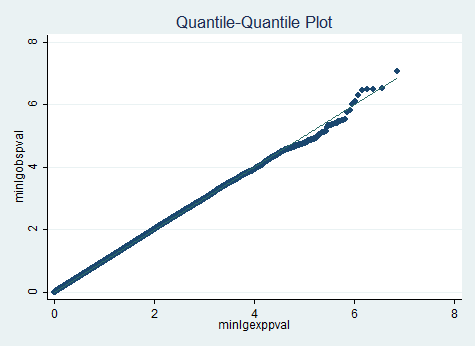

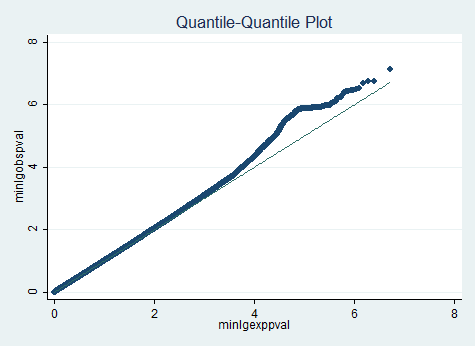

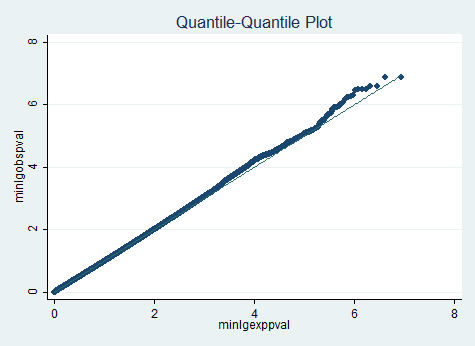


**Supplementary Tables**

**Supplementary Table I:** Top SNPs (P < 1 x 10^-5^) after meta-analysis for CAD association in 4 studies (SCHS, SCADGENS/SP2, SCADGENS/SCES, SCADGENS/SiMES as the discovery datasets)

| NO | SNP | Chr | Pos  (Build 37) | Locus | EA | Meta_P | OR | SE | P_het | P_SCHS | P_SCADGENS/SP2 | P_SCADGENS/SCES | P_SCADGENS/SiMES |
| --- | --- | --- | --- | --- | --- | --- | --- | --- | --- | --- | --- | --- | --- |
| 1 | rs2075291 | 11 | 116661392 | *APOA5* | A | **1.10 x 10^-8^** | 1.587 | 0.081 | 0.776 | 0.001 | 0.003 | 0.028 | 0.003 |
| 2 | rs10738609 | 9 | 22114495 | *CDKN2B-AS1* | G | 8.46 x 10^-8^ | 1.226 | 0.038 | 0.701 | 0.030 | 0.001 | 0.007 | 0.016 |
| 3 | rs141014318 | 9 | 22092924 | *CDKN2B-AS1* | G | 1.10 x 10^-7^ | 1.226 | 0.038 | 0.717 | 0.034 | 0.001 | 0.007 | 0.010 |
| 4 | rs2891168 | 9 | 22098619 | *CDKN2B-AS1* | G | 1.31 x 10^-7^ | 1.222 | 0.038 | 0.844 | 0.022 | 0.001 | 0.010 | 0.011 |
| 5 | rs1537372 | 9 | 22103183 | *CDKN2B-AS1* | T | 1.50 x 10^-7^ | 1.221 | 0.038 | 0.768 | 0.026 | 0.001 | 0.010 | 0.019 |
| 6 | rs4977574 | 9 | 22098574 | *CDKN2B-AS1* | G | 1.50 x 10^-7^ | 1.221 | 0.038 | 0.823 | 0.025 | 0.001 | 0.009 | 0.010 |
| 7 | rs75198898 | 11 | 116649806 | *ZPR1* | A | 1.74 x 10^-7^ | 1.521 | 0.080 | 0.630 | 0.003 | 0.005 | 0.083 | 0.003 |
| 8 | rs3741297 | 11 | 116657667 | *ZPR1* | T | 1.81 x 10^-7^ | 1.524 | 0.081 | 0.598 | 0.003 | 0.004 | 0.098 | 0.002 |
| 9 | rs10757274 | 9 | 22096055 | *CDKN2B-AS1* | G | 1.89 x 10^-7^ | 1.220 | 0.038 | 0.796 | 0.029 | 0.001 | 0.009 | 0.013 |
| 10 | rs113932726 | 11 | 116650638 | *ZPR1* | T | 2.21 x 10^-7^ | 1.516 | 0.080 | 0.633 | 0.003 | 0.005 | 0.080 | 0.003 |
| 11 | rs10757275 | 9 | 22106225 | *CDKN2B-AS1* | A | 2.31 x 10^-7^ | 1.218 | 0.038 | 0.834 | 0.023 | 0.001 | 0.016 | 0.014 |
| 12 | rs2383206 | 9 | 22115026 | *CDKN2B-AS1* | G | 3.72 x 10^-7^ | 1.213 | 0.038 | 0.759 | 0.037 | 0.001 | 0.014 | 0.017 |
| 13 | rs944797 | 9 | 22115286 | *CDKN2B-AS1* | C | 4.59 x 10^-7^ | 1.211 | 0.038 | 0.791 | 0.033 | 0.001 | 0.014 | 0.023 |
| 14 | rs1537376 | 9 | 22116220 | *CDKN2B-AS1* | C | 4.79 x 10^-7^ | 1.211 | 0.038 | 0.792 | 0.033 | 0.001 | 0.014 | 0.023 |
| 15 | kgp2510468 | 12 | 29798058 | *TMTC1* | C | 5.57 x 10^-7^ | 0.733 | 0.062 | 0.783 | 0.004 | 0.002 | 0.148 | 0.011 |
| 16 | rs73073468 | 12 | 29801781 | *TMTC1* | A | 1.21 x 10^-6^ | 0.736 | 0.063 | 0.753 | 0.002 | 0.004 | 0.194 | 0.018 |
| 17 | rs60832006 | 12 | 125368825 | *LOC105370051* | C | 1.22 x 10^-6^ | 1.238 | 0.044 | 0.842 | 0.005 | 0.001 | 0.029 | 0.210 |
| 18 | rs12817233 | 12 | 29800672 | *TMTC1* | G | 1.38 x 10^-6^ | 0.738 | 0.063 | 0.760 | 0.002 | 0.004 | 0.195 | 0.021 |
| 19 | rs77720950 | 12 | 125376496 | *LOC105370051* | T | 1.38 x 10^-6^ | 1.235 | 0.044 | 0.849 | 0.004 | 0.002 | 0.034 | 0.211 |
| 20 | rs12833890 | 12 | 29799526 | *TMTC1* | T | 1.45 x 10^-6^ | 0.738 | 0.063 | 0.763 | 0.003 | 0.004 | 0.195 | 0.021 |
| 21 | rs12814888 | 12 | 29800197 | *TMTC1* | T | 1.45 x 10^-6^ | 0.738 | 0.063 | 0.763 | 0.003 | 0.004 | 0.195 | 0.021 |
| 22 | rs12815417 | 12 | 29800292 | *TMTC1* | A | 1.46 x 10^-6^ | 0.738 | 0.063 | 0.763 | 0.003 | 0.004 | 0.195 | 0.021 |
| 23 | rs56035304 | 12 | 29805197 | *TMTC1* | C | 1.50 x 10^-6^ | 0.740 | 0.063 | 0.810 | 0.003 | 0.004 | 0.165 | 0.031 |
| 24 | rs7859727 | 9 | 22102165 | *CDKN2B-AS1* | T | 1.51 x 10^-6^ | 1.211 | 0.040 | **0.040** | 0.674 | 0.001 | 0.001 | 0.003 |
| 25 | kgp6475705 | 12 | 29798756 | *TMTC1* | T | 1.51 x 10^-6^ | 0.740 | 0.063 | 0.773 | 0.003 | 0.003 | 0.187 | 0.032 |
| 26 | rs7133195 | 12 | 125365645 | *Intergenic* | A | 1.51 x 10^-6^ | 0.808 | 0.044 | 0.866 | 0.007 | 0.002 | 0.019 | 0.209 |
| 27 | rs144858577 | 9 | 22093299 | *CDKN2B-AS1* | T | 1.55 x 10^-6^ | 1.212 | 0.040 | **0.047** | 0.628 | 0.001 | 0.001 | 0.005 |
| 28 | rs1333042 | 9 | 22103813 | *CDKN2B-AS1* | G | 1.58 x 10^-6^ | 1.210 | 0.040 | 0.054 | 0.599 | 0.001 | 0.001 | 0.004 |
| 29 | rs10511701 | 9 | 22112599 | *CDKN2B-AS1* | C | 1.60 x 10^-6^ | 1.210 | 0.040 | 0.069 | 0.556 | 0.001 | 0.001 | 0.004 |
| 30 | rs7857118 | 9 | 22124140 | *CDKN2B-AS1* | T | 1.60 x 10^-6^ | 1.212 | 0.040 | 0.071 | 0.547 | 0.001 | 0.001 | 0.004 |
| 31 | rs1537373 | 9 | 22103341 | *CDKN2B-AS1* | G | 1.62 x 10^-6^ | 1.210 | 0.040 | **0.049** | 0.633 | 0.001 | 0.001 | 0.004 |
| 32 | rs1537371 | 9 | 22099568 | *CDKN2B-AS1* | A | 1.70 x 10^-6^ | 1.210 | 0.040 | **0.040** | 0.689 | 0.001 | 0.001 | 0.003 |
| 33 | rs1556516 | 9 | 22100176 | *CDKN2B-AS1* | C | 1.72 x 10^-6^ | 1.210 | 0.040 | **0.040** | 0.687 | 0.001 | 0.001 | 0.003 |
| 34 | rs1537375 | 9 | 22116071 | *CDKN2B-AS1* | C | 1.72 x 10^-6^ | 1.209 | 0.040 | 0.074 | 0.547 | 0.001 | 0.001 | 0.004 |
| 35 | rs35162187 | 12 | 29800018 | *TMTC1* | T | 1.72 x 10^-6^ | 0.740 | 0.063 | 0.782 | 0.003 | 0.004 | 0.186 | 0.020 |
| 36 | rs10738608 | 9 | 22094796 | *CDKN2B-AS1* | C | 1.79 x 10^-6^ | 1.211 | 0.040 | **0.047** | 0.638 | 0.001 | 0.001 | 0.004 |
| 37 | rs77412840 | 12 | 125373258 | *LOC105370051* | A | 1.81 x 10^-6^ | 1.233 | 0.044 | 0.868 | 0.004 | 0.002 | 0.032 | 0.219 |
| 38 | rs77597682 | 12 | 125368869 | *LOC105370051* | A | 1.83 x 10^-6^ | 1.234 | 0.044 | 0.856 | 0.005 | 0.002 | 0.034 | 0.217 |
| 39 | rs76375378 | 12 | 125368550 | *LOC105370051* | T | 1.84 x 10^-6^ | 1.234 | 0.044 | 0.856 | 0.005 | 0.002 | 0.034 | 0.217 |
| 40 | rs117843422 | 4 | 169205132 | *DDX60* | C | 1.85 x 10^-6^ | 1.369 | 0.066 | 0.304 | 0.002 | 0.202 | 0.001 | 0.062 |
| 41 | rs79758065 | 12 | 125371890 | *LOC105370051* | A | 1.85 x 10^-6^ | 1.233 | 0.044 | 0.869 | 0.004 | 0.002 | 0.032 | 0.218 |
| 42 | rs7341786 | 9 | 22112241 | *CDKN2B-AS1* | C | 1.93 x 10^-6^ | 1.208 | 0.040 | 0.081 | 0.530 | 0.001 | 0.001 | 0.005 |
| 43 | rs7866503 | 9 | 22091924 | *CDKN2B-AS1* | T | 1.96 x 10^-6^ | 1.210 | 0.040 | **0.043** | 0.665 | 0.001 | 0.001 | 0.004 |
| 44 | rs9651879 | 12 | 125380404 | *LOC105370051* | A | 1.97 x 10^-6^ | 1.236 | 0.044 | 0.977 | 0.008 | 0.006 | 0.015 | 0.113 |
| 45 | rs4977757 | 9 | 22094330 | *CDKN2B-AS1* | G | 2.01 x 10^-6^ | 1.210 | 0.040 | 0.051 | 0.628 | 0.001 | 0.001 | 0.004 |
| 46 | rs146564220 | 4 | 169161660 | *DDX60* | T | 2.01 x 10^-6^ | 1.389 | 0.069 | 0.154 | 0.002 | 0.313 | 0.000 | 0.084 |
| 47 | rs76504068 | 12 | 125378781 | *LOC105370051* | T | 2.08 x 10^-6^ | 1.237 | 0.045 | 0.945 | 0.004 | 0.006 | 0.021 | 0.172 |
| 48 | rs74368849 | 11 | 116622299 | *BUD13* | A | 2.11 x 10^-6^ | 1.478 | 0.082 | 0.519 | 0.038 | 0.008 | 0.050 | 0.002 |
| 49 | rs7341791 | 9 | 22112427 | *CDKN2B-AS1* | G | 2.21 x 10^-6^ | 1.207 | 0.040 | 0.076 | 0.556 | 0.001 | 0.001 | 0.005 |
| 50 | rs1831733 | 9 | 22076071 | *CDKN2B-AS1* | C | 2.30 x 10^-6^ | 1.197 | 0.038 | 0.196 | 0.248 | 0.009 | 0.011 | 0.000 |
| 51 | rs2383207 | 9 | 22115959 | *CDKN2B-AS1* | G | 2.35 x 10^-6^ | 1.206 | 0.040 | 0.080 | 0.548 | 0.001 | 0.001 | 0.005 |
| 52 | rs1004638 | 9 | 22115589 | *CDKN2B-AS1* | T | 2.36 x 10^-6^ | 1.206 | 0.040 | 0.081 | 0.547 | 0.001 | 0.001 | 0.005 |
| 53 | rs1537374 | 9 | 22116046 | *CDKN2B-AS1* | G | 2.43 x 10^-6^ | 1.206 | 0.040 | 0.082 | 0.548 | 0.001 | 0.001 | 0.005 |
| 54 | rs4765630 | 12 | 125354892 | *LOC105370050* | A | 2.43 x 10^-6^ | 0.810 | 0.045 | 0.849 | 0.008 | 0.002 | 0.032 | 0.223 |
| 55 | rs11057873 | 12 | 125354885 | *LOC105370050* | C | 2.46 x 10^-6^ | 1.234 | 0.045 | 0.848 | 0.008 | 0.002 | 0.032 | 0.225 |
| 56 | rs2386071 | 5 | 82052001 | *Intergenic* | T | 2.47 x 10^-6^ | 0.831 | 0.039 | 0.825 | 0.003 | 0.051 | 0.004 | 0.112 |
| 57 | rs10757270 | 9 | 22072719 | *CDKN2B-AS1* | G | 2.49 x 10^-6^ | 1.196 | 0.038 | 0.254 | 0.228 | 0.007 | 0.011 | 0.001 |
| 58 | rs74353370 | 12 | 125377282 | *LOC105370051* | A | 2.49 x 10^-6^ | 1.235 | 0.045 | 0.923 | 0.004 | 0.006 | 0.024 | 0.200 |
| 59 | rs1333043 | 9 | 22106731 | *CDKN2B-AS1* | A | 2.51 x 10^-6^ | 1.206 | 0.040 | 0.075 | 0.568 | 0.001 | 0.001 | 0.004 |
| 60 | rs4077220 | 12 | 125359444 | *LOC105370050* | A | 2.53 x 10^-6^ | 0.810 | 0.045 | 0.776 | 0.005 | 0.002 | 0.037 | 0.312 |
| 61 | rs9632885 | 9 | 22072638 | *CDKN2B-AS1* | A | 2.55 x 10^-6^ | 1.197 | 0.038 | 0.285 | 0.199 | 0.009 | 0.012 | 0.001 |
| 62 | rs76718586 | 3 | 184404028 | *Intergenic* | A | 2.66 x 10^-6^ | 1.394 | 0.071 | 0.716 | 0.014 | 0.025 | 0.057 | 0.003 |
| 63 | rs1412834 | 9 | 22110131 | *CDKN2B-AS1* | C | 2.70 x 10^-6^ | 1.206 | 0.040 | 0.074 | 0.576 | 0.001 | 0.001 | 0.004 |
| 64 | rs10738607 | 9 | 22088094 | *CDKN2B-AS1* | G | 2.73 x 10^-6^ | 1.207 | 0.040 | **0.023** | 0.831 | 0.000 | 0.001 | 0.004 |
| 65 | rs10738606 | 9 | 22088090 | *CDKN2B-AS1* | T | 2.73 x 10^-6^ | 1.207 | 0.040 | **0.023** | 0.831 | 0.000 | 0.001 | 0.004 |
| 66 | rs9644861 | 9 | 22090935 | *CDKN2B-AS1* | T | 2.75 x 10^-6^ | 1.208 | 0.040 | 0.062 | 0.599 | 0.002 | 0.001 | 0.005 |
| 67 | rs9644862 | 9 | 22090936 | *CDKN2B-AS1* | G | 2.80 x 10^-6^ | 1.208 | 0.040 | 0.063 | 0.599 | 0.002 | 0.001 | 0.005 |
| 68 | rs6475609 | 9 | 22106271 | *CDKN2B-AS1* | G | 2.94 x 10^-6^ | 1.205 | 0.040 | 0.079 | 0.569 | 0.001 | 0.001 | 0.005 |
| 69 | rs421463 | 14 | 30445235 | *Intergenic* | A | 2.96 x 10^-6^ | 1.230 | 0.044 | **0.018** | 0.000 | 0.827 | 0.000 | 0.182 |
| 70 | rs73141362 | 5 | 82047274 | *Intergenic* | T | 3.01 x 10^-6^ | 0.831 | 0.040 | 0.805 | 0.004 | 0.068 | 0.004 | 0.099 |
| 71 | rs10811653 | 9 | 22091069 | *CDKN2B-AS1* | T | 3.03 x 10^-6^ | 1.206 | 0.040 | **0.033** | 0.766 | 0.001 | 0.001 | 0.004 |
| 72 | rs4765635 | 12 | 125358428 | *LOC105370050* | A | 3.07 x 10^-6^ | 0.813 | 0.044 | 0.870 | 0.008 | 0.002 | 0.036 | 0.213 |
| 73 | rs225919 | 14 | 30442806 | *Intergenic* | T | 3.15 x 10^-6^ | 1.230 | 0.044 | **0.020** | 0.000 | 0.806 | 0.000 | 0.194 |
| 74 | rs116553468 | 4 | 169187080 | *DDX60* | G | 3.30 x 10^-6^ | 1.360 | 0.066 | 0.190 | 0.001 | 0.335 | 0.000 | 0.077 |
| 75 | rs4682867 | 3 | 42905465 | *ACKR2* | T | 3.31 x 10^-6^ | 0.836 | 0.039 | 0.587 | 0.138 | 0.013 | 0.006 | 0.006 |
| 76 | rs149155402 | 5 | 29161970 | *LOC101929660* | G | 3.34 x 10^-6^ | 2.228 | 0.172 | 0.794 | 0.019 | 0.002 | 0.062 | 0.035 |
| 77 | rs225916 | 14 | 30443903 | *Intergenic* | G | 3.45 x 10^-6^ | 1.228 | 0.044 | **0.022** | 0.000 | 0.783 | 0.000 | 0.212 |
| 78 | rs4450249 | 12 | 125355418 | *LOC105370050* | T | 3.45 x 10^-6^ | 1.230 | 0.045 | 0.851 | 0.008 | 0.002 | 0.040 | 0.227 |
| 79 | rs10846761 | 12 | 125354164 | *LOC105370050* | C | 3.51 x 10^-6^ | 0.813 | 0.045 | 0.847 | 0.008 | 0.002 | 0.043 | 0.226 |
| 80 | rs225906 | 14 | 30452968 | *Intergenic* | G | 3.53 x 10^-6^ | 1.228 | 0.044 | **0.023** | 0.000 | 0.773 | 0.000 | 0.213 |
| 81 | rs7859362 | 9 | 22105927 | *CDKN2B-AS1* | C | 3.64 x 10^-6^ | 1.203 | 0.040 | 0.076 | 0.596 | 0.001 | 0.001 | 0.006 |
| 82 | rs2235716 | 6 | 1607116 | *FOXCUT* | C | 3.68 x 10^-6^ | 1.203 | 0.040 | 0.966 | 0.016 | 0.023 | 0.047 | 0.009 |
| 83 | rs80321171 | 12 | 125362473 | *LOC105370050* | C | 3.80 x 10^-6^ | 1.228 | 0.044 | 0.806 | 0.005 | 0.002 | 0.040 | 0.306 |
| 84 | rs10757273 | 9 | 22090301 | *CDKN2B-AS1* | A | 4.04 x 10^-6^ | 1.203 | 0.040 | **0.042** | 0.722 | 0.001 | 0.001 | 0.005 |
| 85 | kgp12301589 | 19 | 38735581 | *Intergenic* | A | 4.09 x 10^-6^ | 1.518 | 0.091 | 0.571 | 0.038 | 0.007 | 0.087 | 0.003 |
| 86 | rs9644860 | 9 | 22090603 | *CDKN2B-AS1* | T | 4.10 x 10^-6^ | 1.203 | 0.040 | **0.036** | 0.770 | 0.001 | 0.001 | 0.004 |
| 87 | rs9644859 | 9 | 22090521 | *CDKN2B-AS1* | A | 4.13 x 10^-6^ | 1.203 | 0.040 | **0.036** | 0.772 | 0.001 | 0.001 | 0.004 |
| 88 | rs225918 | 14 | 30443654 | *Intergenic* | A | 4.22 x 10^-6^ | 1.225 | 0.044 | **0.020** | 0.000 | 0.732 | 0.000 | 0.284 |
| 89 | rs4765636 | 12 | 125359899 | *LOC105370050* | G | 4.24 x 10^-6^ | 0.815 | 0.044 | 0.616 | 0.004 | 0.002 | 0.029 | 0.511 |
| 90 | rs225908 | 14 | 30449594 | *Intergenic* | A | 4.26 x 10^-6^ | 1.225 | 0.044 | **0.019** | 0.000 | 0.798 | 0.000 | 0.246 |
| 91 | rs170475 | 14 | 30451426 | *Intergenic* | C | 4.28 x 10^-6^ | 1.226 | 0.044 | **0.019** | 0.000 | 0.796 | 0.000 | 0.246 |
| 92 | rs75669656 | 12 | 29809843 | *TMTC1* | T | 4.39 x 10^-6^ | 0.707 | 0.076 | 0.860 | 0.034 | 0.003 | 0.029 | 0.061 |
| 93 | rs16934692 | 12 | 29811471 | *TMTC1* | A | 4.43 x 10^-6^ | 0.744 | 0.064 | 0.676 | 0.002 | 0.010 | 0.285 | 0.021 |
| 94 | rs10757272 | 9 | 22088260 | *CDKN2B-AS1* | T | 4.49 x 10^-6^ | 1.202 | 0.040 | **0.021** | 0.903 | 0.001 | 0.001 | 0.004 |
| 95 | kgp8467651 | 12 | 29813972 | *TMTC1* | T | 4.64 x 10^-6^ | 0.708 | 0.075 | 0.891 | 0.030 | 0.003 | 0.029 | 0.059 |
| 96 | rs12517454 | 5 | 82052937 | *Intergenic* | A | 4.89 x 10^-6^ | 0.836 | 0.039 | 0.790 | 0.006 | 0.052 | 0.004 | 0.144 |
| 97 | rs11057912 | 12 | 125411748 | *Intergenic* | T | 5.34 x 10^-6^ | 1.221 | 0.044 | 0.802 | 0.007 | 0.002 | 0.136 | 0.140 |
| 98 | rs10865924 | 3 | 42894427 | *ACKR2* | G | 5.60 x 10^-6^ | 0.841 | 0.038 | 0.681 | 0.130 | 0.005 | 0.014 | 0.015 |
| 99 | rs143618462 | 3 | 42904488 | *ACKR2* | G | 5.71 x 10^-6^ | 0.841 | 0.038 | 0.680 | 0.131 | 0.005 | 0.014 | 0.015 |
| 100 | rs148198222 | 3 | 42901977 | *ACKR2* | C | 5.73 x 10^-6^ | 0.841 | 0.038 | 0.681 | 0.131 | 0.005 | 0.014 | 0.015 |
| 101 | rs2099022 | 3 | 42892048 | *ACKR2* | A | 5.77 x 10^-6^ | 0.841 | 0.038 | 0.683 | 0.130 | 0.005 | 0.013 | 0.015 |
| 102 | rs74504220 | 3 | 42903920 | *ACKR2* | G | 5.98 x 10^-6^ | 0.841 | 0.038 | 0.683 | 0.131 | 0.005 | 0.014 | 0.016 |
| 103 | rs4682866 | 3 | 42903349 | *ACKR2* | T | 5.99 x 10^-6^ | 0.841 | 0.038 | 0.683 | 0.131 | 0.005 | 0.014 | 0.016 |
| 104 | rs17030478 | 3 | 42893486 | *ACKR2* | G | 6.03 x 10^-6^ | 0.841 | 0.038 | 0.686 | 0.130 | 0.005 | 0.014 | 0.016 |
| 105 | rs12635458 | 3 | 42891616 | *ACKR2* | G | 6.04 x 10^-6^ | 0.841 | 0.038 | 0.685 | 0.130 | 0.005 | 0.013 | 0.016 |
| 106 | rs11129980 | 3 | 42895982 | *ACKR2* | T | 6.06 x 10^-6^ | 0.841 | 0.038 | 0.686 | 0.130 | 0.005 | 0.014 | 0.016 |
| 107 | rs4683344 | 3 | 42884204 | *ACKR2* | C | 6.19 x 10^-6^ | 0.841 | 0.038 | 0.681 | 0.133 | 0.006 | 0.013 | 0.016 |
| 108 | rs4683342 | 3 | 42877433 | *ACKR2* | C | 6.48 x 10^-6^ | 0.842 | 0.038 | 0.676 | 0.136 | 0.006 | 0.012 | 0.017 |
| 109 | rs1537370 | 9 | 22084310 | *CDKN2B-AS1* | T | 6.54 x 10^-6^ | 1.203 | 0.041 | **0.002** | 0.708 | 0.002 | 0.002 | 0.000 |
| 110 | rs1427803 | 3 | 42911723 | *Intergenic* | A | 6.54 x 10^-6^ | 0.841 | 0.038 | 0.653 | 0.149 | 0.006 | 0.016 | 0.011 |
| 111 | rs6475608 | 9 | 22101702 | *CDKN2B-AS1* | C | 6.57 x 10^-6^ | 1.200 | 0.041 | 0.066 | 0.647 | 0.001 | 0.001 | 0.010 |
| 112 | rs13068402 | 3 | 42875756 | *ACKR2* | C | 7.03 x 10^-6^ | 0.842 | 0.038 | 0.677 | 0.137 | 0.006 | 0.012 | 0.018 |
| 113 | rs34759881 | 3 | 42875544 | *ACKR2* | G | 7.08 x 10^-6^ | 0.842 | 0.038 | 0.677 | 0.137 | 0.006 | 0.012 | 0.018 |
| 114 | rs4077219 | 12 | 125359627 | *LOC105370050* | A | 7.28 x 10^-6^ | 0.820 | 0.044 | 0.722 | 0.010 | 0.001 | 0.052 | 0.316 |
| 115 | rs4682862 | 3 | 42877413 | *ACKR2* | G | 7.57 x 10^-6^ | 0.839 | 0.039 | 0.592 | 0.122 | 0.014 | 0.002 | 0.039 |
| 116 | rs9951590 | 18 | 74330117 | *LINC00683* | C | 8.05 x 10^-6^ | 1.375 | 0.071 | 0.513 | 0.024 | 0.166 | 0.256 | 0.000 |
| 117 | rs9948412 | 18 | 74329454 | *Intergenic* | C | 8.06 x 10^-6^ | 1.374 | 0.071 | 0.512 | 0.024 | 0.166 | 0.256 | 0.000 |
| 118 | rs9956431 | 18 | 74329251 | *Intergenic* | T | 8.09 x 10^-6^ | 1.374 | 0.071 | 0.513 | 0.024 | 0.166 | 0.257 | 0.000 |
| 119 | rs9959410 | 18 | 74329953 | *Intergenic* | A | 8.13 x 10^-6^ | 1.374 | 0.071 | 0.512 | 0.024 | 0.167 | 0.256 | 0.000 |
| 120 | rs937898 | 18 | 74330672 | *LINC00683* | G | 8.59 x 10^-6^ | 1.380 | 0.072 | 0.531 | 0.026 | 0.155 | 0.250 | 0.000 |
| 121 | rs9959129 | 18 | 74329661 | *Intergenic* | A | 8.97 x 10^-6^ | 1.372 | 0.071 | 0.515 | 0.026 | 0.175 | 0.243 | 0.000 |
| 122 | rs9405496 | 6 | 1608631 | *Intergenic* | C | 9.00 x 10^-6^ | 0.824 | 0.044 | 0.467 | 0.119 | 0.003 | 0.135 | 0.003 |
| 123 | rs138419570 | 3 | 184404582 | Intergenic | A | 9.95 x 10^-6^ | 1.358 | 0.069 | 0.633 | 0.034 | 0.033 | 0.051 | 0.004 |

EA: Effect allele; OR: odds ratio; CI: confidence interval; P_het: P values for Cochran’s Q.

**Supplementary Table II:** Associations of rs2075291 on lipid levels in SCHS and SP2 datasets

|  | SCHS | | | | |  | SP2 | | | | |
| --- | --- | --- | --- | --- | --- | --- | --- | --- | --- | --- | --- |
| Outcome | N | EAF | Beta | SE | P |  | N | EAF | Beta | SE | P |
| HDL_z | 1978 | 0.061 | -0.196 | 0.062 | **0.002** |  | 2157 | 0.057 | -0.401 | 0.059 | **9.76 x 10^-12^** |
| LDL_z | 1978 | 0.061 | -0.032 | 0.066 | 0.629 |  | 2140 | 0.057 | 0.008 | 0.064 | 0.895 |
| TG_z | 1978 | 0.061 | 0.226 | 0.069 | **0.001** |  | 2156 | 0.057 | 0.392 | 0.061 | **9.80 x 10^-11^** |
| CHOL_z | 1891 | 0.061 | 0.008 | 0.065 | 0.905 |  | 2157 | 0.057 | 0.015 | 0.063 | 0.808 |
| HDL_z adj for TG_z | 1978 | 0.061 | -0.077 | 0.059 | 0.191 |  | 2156 | 0.057 | -0.225 | 0.053 | **2.34 x 10^-5^** |
| TG_z adj for HDL_z | 1978 | 0.061 | 0.146 | 0.062 | **0.018** |  | 2156 | 0.057 | 0.208 | 0.055 | **1.45 x 10^-4^** |

EAF: Effect allele frequency.

**Supplementary Table III:** Clinical characteristics of 5 datasets used in this study

|  | | SCHS | | | SCADGENS/SP2 | | | | | SCADGENS/SCES | | SCADGENS/SiMES | | | SCADGENS/SINDI | | |  |
| --- | --- | --- | --- | --- | --- | --- | --- | --- | --- | --- | --- | --- | --- | --- | --- | --- | --- | --- |
|  | Case | | Control | P | | OR | Case | Control | Case | | Control | | Case | Control | | Case | Control | |
| Sample size | 718 | | 1262 | -- | | -- | 429 | 2189 | 631 | | 1713 | | 391 | 2212 | | 291 | 1848 | |
| Percent of men (%) | 64.6 | | 63.2 | -- | | -- | 88.1 | 45.9 | 96.5 | | 50.2 | | 88.3 | 52.1 | | 87.3 | 50.7 | |
| Age (mean/SD) | 66.6(7.8) | | 66.2(7.8) | -- | | -- | 56.0(9.8) | 47.8(11.1) | 57.8(8.2) | | 57.5(11.1) | | 55.9(7.7) | 58.4(11.0) | | 55.0(9.0) | 57.1(9.8) | |
| HDL (mmol/L) (mean/SD) | 1.28 (0.30) | | 1.35 (0.33) | **7.02 x 10^-6^** | | 0.50 | NA | 1.47 (0.36) | NA | | NA | | NA | NA | | NA | NA | |
| LDL (mmol/L) (mean/SD) | 3.34 (0.87) | | 3.17 (0.80) | **2.71 x 10^-5^** | | 1.27 | NA | 3.14 (0.82) | NA | | NA | | NA | NA | | NA | NA | |
| TG (mmol/L) (mean/SD) | 1.67 (0.69) | | 1.57 (0.68) | **0.001** | | 1.27 | NA | 1.27 (0.71) | NA | | NA | | NA | NA | | NA | NA | |
| TC (mmol/L) (mean/SD) | 5.31 (1.01) | | 5.18 (0.90) | **0.002** | | 1.17 | NA | 5.20 (0.92) | NA | | NA | | NA | NA | | NA | NA | |
| BMI (kg/m^2^) (mean/SD) | 23.25 (3.48) | | 22.83 (3.27) | **0.025** | | 1.04 | NA | 22.80 (3.70) | NA | | NA | | NA | NA | | NA | NA | |
| SBP (mean/SD) | 149.52 (24.34) | | 140.03 (21.56) | **1.94 x 10^-16^** | | 1.02 | NA | 128.96 (19.59) | NA | | NA | | NA | NA | | NA | NA | |
| DBP (mean/SD) | 83.50 (14.35) | | 80.60 (10.49) | **1.99 x 10^-6^** | | 1.02 | NA | 76.74 (10.79) | NA | | NA | | NA | NA | | NA | NA | |

OR: odds ratio.

**Supplementary Table IV:** Associations of rs2075291 on CAD status in SCHS dataset

|  | SCHS | | | | |
| --- | --- | --- | --- | --- | --- |
| Outcome | N (cases/controls) | EAF | OR | 95% CI | P |
| CAD | 718 / 1262 | 0.061 | 1.552 | 1.192 - 2.019 | **0.001** |
| CAD adjusted for TG_z | 718 / 1260 | 0.061 | 1.591 | 1.206 - 2.099 | **0.001** |
| CAD adjusted for HDL_z | 718 / 1260 | 0.061 | 1.490 | 1.143 - 1.942 | **0.003** |
| CAD adjusted for all lipids | 682 / 1209 | 0.061 | 1.580 | 1.194 - 2.090 | **0.001** |
| CAD adjusted for lipids, BMI, BP, HbA1c | 492 / 904 | 0.061 | 1.581 | 1.140 - 2.194 | **0.006** |

EAF: Effect allele frequency; OR: odds ratio; CI: confidence interval.

**Supplementary Table V:** 49 genes for SNPs that were in at least moderate LD with rs2075291 and the 11 index SNPs showing suggestive and significant associations in our study (r^2^ > 0.2 in 1000G ASN populations)

| Index SNP | Locus | Chr | Pos  (Build 37) | Genes in LD with index SNP |
| --- | --- | --- | --- | --- |
| rs12202017 | *TCF21* | 6 | 134173151 | *EYA4, TCF21, LINC01312* |
| rs12413409 | *CYP17A1-CNNM2-NT5C2* | 10 | 104719096 | *RPARP−AS1, C10orf95, TMEM180, ACTR1A, SUFU, TRIM8, ARL3, WBP1L, C10orf32, ASMT, SFXN2, CYP17A1, C10orf32, CNNM2, AS3MT, NT5C2, INA, PDCD11, RPEL1, CALHM2, PCGF6, TAF5, USMG5, MIR1307, CALHM1, CALHM3, LOC102724341* |
| rs17411031 | *LPL* | 8 | 19852310 | *LPL* |
| rs2075650 | *APOE-APOC1* | 19 | 45395619 | *APOE, APOC4, PVRL2, TOMM40, APOC1,* |
| rs4977574 | *9p21* | 9 | 22098574 | *CDKN2A, CDKN2B, CDKN2B-AS1, MTAP* |
| rs532436 | *ABO* | 9 | 136149830 | *ABO* |
| rs663129 | *PMAIP1-MC4R* | 18 | 57838401 | *MC4R* |
| rs6903956 | *ADTRP-C6orf105* | 6 | 11774583 | *ADTRP* |
| rs7173743 | *ADAMTS7* | 15 | 79141784 | *CTSH, RASGRF1, MORF4L1, ADAMTS7, LOC646938* |
| rs9349379 | *PHACTR1* | 6 | 12903957 | *PHACTR1* |
| rs974819 | *PDGFD* | 11 | 103660567 | *PDGFD, MIR4693* |
| rs2075291 | *APOA5* | 11 | 116661392 | *BUD13, ZPR1, APOA5, APOA4,* |

**Supplementary Table VI:** Canonical pathways for all loci that showed an association in our study using Ingenuity Pathway Analysis

| Ingenuity Canonical Pathways | B-H P-value | Ratio | Molecules |
| --- | --- | --- | --- |
| LXR/RXR Activation | **1.10 x 10^-5^** | 4.96 x 10^-2^ | *APOE, APOA4, APOC4, LPL, APOC1, APOA5* |
| Atherosclerosis Signaling | **1.10 x 10^-5^** | 4.72 x 10^-2^ | *APOE, APOA4, APOC4, LPL, APOC1, PDGFD* |
| FXR/RXR Activation | **1.82 x 10^-4^** | 3.97 x 10^-2^ | *APOE, APOA4, APOC4, LPL, APOC1* |
| Clathrin-mediated Endocytosis Signaling | **1.17 x 10^-3^** | 2.54 x 10^-2^ | *APOE, APOA4, APOC4, APOC1, PDGFD* |
| IL-12 Signaling and Production in Macrophages | **4.07 x 10^-3^** | 2.74 x 10^-2^ | *APOE, APOA4, APOC4, APOC1* |
| Production of Nitric Oxide and Reactive Oxygen Species in Macrophages | **9.77 x 10^-3^** | 2.07 x 10^-2^ | *APOE, APOA4, APOC4, APOC1* |
| Glioma Signaling | **1.86 x 10^-2^** | 2.73 x 10^-2^ | *CDKN2A, PDGFD, CDKN2B* |

B-H P-value: P value after Benjamini-Hochberg multiple testing correction

**Supplementary Table VII:** DEPICT Gene Enrichment Sets Based on rs2075291 and 11 SNPs that showed an association in our study (Top 20 nominal P value)

| Original gene set ID | Original gene set description | Nominal P-value |
| --- | --- | --- |
| ENSG00000159199 | ATP5G1 subnetwork | **2.31 x 10^-7^** |
| MP:0001927 | Abnormal estrous cycle | **6.02 x 10^-6^** |
| GO:0050795 | Regulation of behavior | **6.66 x 10^-5^** |
| GO:0008217 | Regulation of blood pressure | **8.18 x 10^-5^** |
| ENSG00000198899 | MT-ATP6 subnetwork | **8.68 x 10^-5^** |
| ENSG00000115718 | PROC subnetwork | **9.86 x 10^-5^** |
| MP:0003954 | Abnormal Reichert's membrane morphology | **1.06 x 10^-4^** |
| GO:0035150 | Regulation of tube size | **1.07 x 10^-4^** |
| GO:0050880 | Regulation of blood vessel size | **1.09 x 10^-4^** |
| REACTOME_SPHINGOLIPID_METABOLISM | REACTOME_SPHINGOLIPID_METABOLISM | **1.15 x 10^-4^** |
| REACTOME_GLYCOSPHINGOLIPID_METABOLISM | REACTOME_GLYCOSPHINGOLIPID_METABOLISM | **1.19 x 10^-4^** |
| MP:0003229 | Abnormal vitelline vasculature morphology | **1.24 x 10^-4^** |
| GO:0045940 | Positive regulation of steroid metabolic process | **1.56 x 10^-4^** |
| KEGG_GLYCOSPHINGOLIPID_BIOSYNTHESIS_GLOBO_SERIES | KEGG_GLYCOSPHINGOLIPID_BIOSYNTHESIS_GLOBO_SERIES | **1.57 x 10^-4^** |
| GO:0003018 | Vascular process in circulatory system | **2.02 x 10^-4^** |
| ENSG00000172380 | GNG12 subnetwork | **2.06 x 10^-4^** |
| ENSG00000121989 | ACVR2A subnetwork | **2.42 x 10^-4^** |
| MP:0005590 | Increased vasodilation | **2.57 x 10^-4^** |
| MP:0001126 | Abnormal ovary morphology | **2.64 x 10^-4^** |
| ENSG00000078401 | EDN1 subnetwork | **2.70 x 10^-4^** |

**Supplementary Table VIII: CAD associations for regional SNPs that were in at least moderate LD with the index SNPs (r^2^ > 0.2 in 1000G ASN populations)**

| Index rsid | Regional rsid | LD | Chr | Locus | Index P | Index P_adj |  | Regional EA | Regional P | Regional P_adj | Regional OR | Regional P_het |
| --- | --- | --- | --- | --- | --- | --- | --- | --- | --- | --- | --- | --- |
|  |
| rs12202017 | rs9375986 | 0.8-1.0 | 6 | *TCF21* | **3.08 x 10^-3^** | 0.142 |  | G | **4.18 x 10^-4^** | **0.019** | 1.112 | 0.321 |
| rs12413409 | rs113278154 | 0.8-1.0 | 10 | *CYP17A1-CNNM2-NT5C2* | **2.12 x 10^-2^** | 0.975 |  | T | **1.25 x 10^-3^** | 0.057 | 0.916 | **0.002** |
| rs974819 | rs2019090 | 0.8-1.0 | 11 | *PDGFD* | **2.52 x 10^-3^** | 0.116 |  | T | **1.55 x 10^-3^** | 0.071 | 0.913 | 0.103 |
| rs6903956 | rs4713894 | 0.8-1.0 | 6 | *ADTRP-C6orf105* | **5.24 x 10^-3^** | 0.241 |  | C | **1.85 x 10^-3^** | 0.085 | 0.770 | **0.038** |
| rs2048327 | rs2063192 | 0.2-0.4 | 6 | *SLC22A3, LPAL2, LPA* | 0.136 | 1.000 |  | G | **4.58 x 10^-3^** | 0.211 | 0.895 | 0.084 |
| rs17411031 | rs11989309 | 0.4-0.6 | 8 | *LPL* | **4.73 x 10^-2^** | 1.000 |  | T | **7.00 x 10^-3^** | 0.322 | 0.843 | 0.110 |
| rs663129 | rs12954782 | 0.6-0.8 | 18 | *PMAIP1, MC4R* | **2.69 x 10^-2^** | 1.000 |  | G | **1.07 x 10^-2^** | 0.494 | 1.121 | 0.105 |
| rs8042271 | rs7182288 | 0.6-0.8 | 15 | *MFGE8-ABHD2* | 6.61 x 10^-2^ | 1.000 |  | T | **1.08 x 10^-2^** | 0.498 | 0.908 | 0.089 |
| rs10953541 | rs75268817 | 0.4-0.6 | 7 | *7q22* | 0.114 | 1.000 |  | A | **1.10 x 10^-2^** | 0.507 | 0.894 | 0.622 |
| rs17087335 | rs10008356 | 0.2-0.4 | 4 | *REST, NOA1* | 0.299 | 1.000 |  | G | **1.85 x 10^-2^** | 0.851 | 0.913 | 0.198 |
| rs216172 | rs36113682 | 0.4-0.6 | 17 | *SMG6* | 0.346 | 1.000 |  | T | **2.15 x 10^-2^** | 0.991 | 0.920 | 0.160 |
| rs9970807 | rs7368386 | 0.4-0.6 | 1 | *PPAP2B* | 0.125 | 1.000 |  | A | **4.11 x 10^-2^** | 1.000 | 1.156 | 0.158 |
| rs7212798 | rs28566846 | 0.8-1.0 | 17 | *BCAS3* | 9.51 x 10^-2^ | 1.000 |  | T | **4.12 x 10^-2^** | 1.000 | 1.123 | 0.520 |
| rs6725887 | rs111485191 | 0.2-0.4 | 2 | *WDR12* | 0.211 | 1.000 |  | T | **4.24 x 10^-2^** | 1.000 | 1.134 | 0.914 |
| rs7692387 | rs10017307 | 0.8-1.0 | 4 | *GUCY1A3* | 9.94 x 10^-2^ | 1.000 |  | T | **4.37 x 10^-2^** | 1.000 | 0.901 | 0.086 |
| rs646776 | rs4970834 | 0.6-0.8 | 1 | *SORT1* | 6.52 x 10^-2^ | 1.000 |  | T | **4.92 x 10^-2^** | 1.000 | 0.913 | 0.079 |
| rs16986953 | rs10189561 | 0.2-0.4 | 2 | *AK097927* | 0.134 | 1.000 |  | C | **4.99 x 10^-2^** | 1.000 | 0.943 | 0.566 |
| rs501120 | rs7894693 | 0.4-0.6 | 10 | *CXCL12* | 0.191 | 1.000 |  | T | 5.58 x 10^-2^ | 1.000 | 0.909 | **0.023** |
| rs4593108 | rs12501429 | 0.2-0.4 | 4 | *EDNRA* | 0.159 | 1.000 |  | C | 6.63 x 10^-2^ | 1.000 | 1.068 | 0.517 |
| rs56336142 | rs9394577 | 0.6-0.8 | 6 | *KCNK5* | 0.194 | 1.000 |  | C | 8.46 x 10^-2^ | 1.000 | 0.943 | 0.821 |
| rs1561198 | rs11311 | 0.8-1.0 | 2 | *VAMP5, VAMP8, GGCX* | 0.137 | 1.000 |  | T | 0.111 | 1.000 | 1.060 | 0.858 |
| rs2107595 | rs2074633 | 0.8-1.0 | 7 | *HDAC9* | 0.164 | 1.000 |  | C | 0.125 | 1.000 | 1.044 | 0.718 |

EA: Effect allele; OR: odds ratio; CI: confidence interval; P_adj : P adjusted for 46 tests. P_het: P values for Cochran’s Q.

**Supplementary Table IX:** Sample QC procedures performed for 5 GWAS datasets

|  | SCHS | SCADGENS/SP2 | SCADGENS/SCES | SCADGENS/SiMES | SCADGENS/SINDI |
| --- | --- | --- | --- | --- | --- |
| Ethnicity | Chinese | Chinese | Chinese | Malays | Indians |
| Total number of samples | 2136 | 2868 | 2464 | 2842 | 2459 |
| Positive controls | 42 | 63 | 0 | 0 | 0 |
| Samples with low call rates (<0.98) | 10 | 12 | 6 | 3 | 2 |
| Extremes in heterozygozity  (< or >3SD) | 20 | 29 | 18 | 3 | 3 |
| Samples from IBD analysis | 25 | 79 | 17 | 60 | 55 |
| Samples from PCA analysis | 36 | 67 | 79 | 174 | 260 |
| Mismatch of case status | 23 | 0 | 0 | 0 | 0 |
| Remaining samples | 718 cases/1262 controls | 429 cases/2189 controls | 631 cases/1713 controls | 391 cases/2212 controls | 291 cases/1848 controls |

**Supplementary Table X:** SNPs quality control filters for 5 GWAS datasets

|  | SCHS | SCADGENS/SP2 | SCADGENS/SCES | SCADGENS/SiMES | SCADGENS/SINDI |
| --- | --- | --- | --- | --- | --- |
| SNP arrays used | Illumina HumanOmniZhonghua8v1 | Illumina HumanOmniZhonghua8v1, Illimina 1Mduo and Illumina 610Quad | Illumina HumanOmniZhonghua8v1 and Illumina 610Quad | Illumina HumanOmniZhonghua8v1 and Illumina 610Quad | Illumina HumanOmniZhonghua8v1 and Illumina 610Quad |
| Strand | forward | forward | forward | forward | forward |
| Genome Build | 37 | 37 | 37 | 37 | 37 |
| Imputation |  |  |  |  |  |
| Program used | IMPUTE 2 | IMPUTE 2 | IMPUTE 2 | IMPUTE 2 | IMPUTE 2 |
| Reference panel | 1000G phase 3 | 1000G phase 3 | 1000G phase 3 | 1000G phase 3 | 1000G phase 3 |
| SNP QC |  |  |  |  |  |
| Overlapping genotyped SNPs | 803001 | 253930 | 266794 | 299488 | 311483 |
| Total number of genotyped and imputed SNPs | 80292860 | 83902620 | 81706806 | 81706898 | 81706929 |
| non SNV variants | 421520 | 425908 | 3887711 | 3887711 | 3887711 |
| MAF < 0.01 | 71737418 | 10204200 | 10199054 | 12369781 | 14489181 |
| HWE P-value<10^-5^ | 1187 | 190 | 360 | 200 | 480 |
| Low quality imputation scores | 2304989 | 66263383 | 60389430 | 57870489 | 55892937 |
| Call rate < 0.95 | 416 | 149 | 136 | 28 | 22 |
| Remaining SNPs | 5827330 | 7008790 | 7230115 | 7578689 | 7436598 |
